# Three-Dimensional Interactions Analysis of the Anticancer Target c-Src Kinase with Its Inhibitors

**DOI:** 10.3390/cancers12082327

**Published:** 2020-08-18

**Authors:** Vibhu Jha, Marco Macchia, Tiziano Tuccinardi, Giulio Poli

**Affiliations:** Department of Pharmacy, University of Pisa, 56126 Pisa, Italy; vibhu.jha@farm.unipi.it (V.J.); marco.macchia@unipi.it (M.M.); giulio.poli@unipi.it (G.P.)

**Keywords:** c-Src kinase, ligand–protein interaction

## Abstract

Src family kinases (SFKs) constitute the biggest family of non-receptor tyrosine kinases considered as therapeutic targets for cancer therapy. An aberrant expression and/or activation of the proto-oncogene c-Src kinase, which is the oldest and most studied member of the family, has long been demonstrated to play a major role in the development, growth, progression and metastasis of numerous human cancers, including colon, breast, gastric, pancreatic, lung and brain carcinomas. For these reasons, the pharmacological inhibition of c-Src activity represents an effective anticancer strategy and a few compounds targeting c-Src, together with other kinases, have been approved as drugs for cancer therapy, while others are currently undergoing preclinical studies. Nevertheless, the development of potent and selective inhibitors of c-Src aimed at properly exploiting this biological target for the treatment of cancer still represents a growing field of study. In this review, the co-crystal structures of c-Src kinase in complex with inhibitors discovered in the past two decades have been described, highlighting the key ligand–protein interactions necessary to obtain high potency and the features to be exploited for addressing selectivity and drug resistance issues, thus providing useful information for the design of new and potent c-Src kinase inhibitors.

## 1. Introduction

Protein kinases (PKs) play an important role in cellular signal transduction pathways and regulate important cellular processes such as cell growth through post-translational phosphorylation. PKs are one of the largest protein families, since the human kinome encodes for 518 protein kinases in total, which corresponds to 1.7% of the whole genome [1]. Protein kinases catalyze the transfer of the γ-phosphate group from ATP to serine, threonine or tyrosine residues of their substrate proteins [2]. Such post-translational modifications provide a mechanism to modulate enzymatic activity or intermolecular interactions of substrate proteins in response to endogenous and exogenous signals. Overexpression or mutations of protein kinases can lead to a variety of human diseases and autoimmunity [3]. Kinases are found to be implicated in the development of many diseases, such as cancer, diabetes and Alzheimer’s disease, and account for the second most exploited group of drug targets, with numerous drug discovery efforts ongoing today and 55 kinase inhibitors approved for the treatment of a variety of cancers in the last two decades [4,5].

Src family kinases (SFKs) is the biggest family of non-receptor tyrosine kinases discovered as major biological targets in a wide variety of human cancers [6]. SFKs can be further subdivided into a group of eight typical members, including c-Src, Yes, Fyn, Fgr, Lck, Hck, Blk and Lyn, and three atypical members represented by Brk, Frk and Srm. [7]. SFKs were reported to be involved in various physiological processes such as cell adhesion, migration, differentiation and apoptosis. Furthermore, SFKs were found to play important roles in different pathological conditions like cancer, hematopoietic disorders, epilepsy, parkinsonism, ischemic preconditioning, ischemic-reperfusion injury, learning and memory. Over the past two decades, the role SFKs in the development of cancer has been extensively studied. For instance, Yes kinase was found to be frequently hyperexpressed in colon and breast cancers, while high level of Fyn kinase were detected in brain cancer. Lyn kinase showed to be implicated in the development of prostate cancer, whereas notable levels of Lck, Hck, Fgr and Blk kinases have been found in hematopoietic cancers [8]. Among all SFKs, c-Src kinase is the oldest and most studied cellular protein kinase, which is aberrantly expressed and/or activated in a broad variety of cancers. c-Src is considered as a key anticancer target and many drug discovery programs aimed at identifying novel kinase inhibitors as anticancer drugs were focused on targeting c-Src. In fact, a significantly high number of X-ray structures of c-Src in complex with small-molecule inhibitors have been reported. For this reason, the present review focused on the three-dimensional interactions analysis of c-Src kinase with its inhibitors.

The c-Src proto-oncogene is reported to play a major role in the development, growth, progression, and metastasis of numerous human cancers [9]. c-Src hyperactivation as a result of increased kinase activity and/or protein expression levels has been found in major types of cancers, including colon, breast, pancreatic, lung and brain carcinomas. More specifically, in human mammary carcinomas, a c-Src kinase activity up to 20-fold higher than normal tissues has been observed. A 30-fold higher c-Src activity has also been found in the cell lines of these breast tumors [10]. The increase of c-Src activity is an early event occurring in premalignant colon tissues; in fact, c-Src kinase activity was found to be increased by fivefold to eightfold in most colon tumors. Increased c-Src protein levels have also been observed in lung carcinoma, adenocarcinoma and gastric cancers, neuroblastomas and pancreatic cancers [11]. One of the proposed mechanisms at the basis of the of high levels of c-Src activation suggests that the kinase activity of c-Src could be increased by direct or indirect interaction with receptor tyrosine kinases like EGFR, PDGFR, FGFR, CSR-1R, HER2 and c-Met, which leads to synergistic levels of tumorigenicity [12]. Another potential mechanism explains the post-translational activation of c-Src, which may involve insufficient Csk kinase activity or increased activity of c-Src phosphatases, leading to the dephosphorylation of the regulatory Y530 of c-Src (*vide infra*) [13]. c-Src has shown to interact with regulatory pathways in the cell cycle and signal transduction cascades involved with cell proliferation. For instance, c-Src activation provides a link with Ras/MAPK cascade and showed to activate the transcription factor STAT3 [14]. All these events in which c-Src is involved, contribute in elevating tumor growth and promote the development of metastatic phenotypes. For these reasons, c-Src has long been considered as a major target for therapeutic intervention in cancer treatment and the mechanistic studies of c-Src function and role in these pathologies have been steadily advanced [15,16]. Nevertheless, c-Src inhibitors tend to have limited selectivity over other SFKs and different kinases because of a high degree of sequence conservation within the kinase domain [17]. Selective chemical tools are still required to inspect the complex nature of kinase regulation despite the extensive research in the field [18]. In this context, the information about the target structure and the fundamental ligand–protein interactions that need to be addressed in order to achieve a high inhibitory activity is a key aspect for the structure-based design of potent and selective inhibitors. Most of c-Src inhibitors display ATP-competitive mechanism of inhibition on the active site of c-Src. The structural features and the overall size of these inhibitors determine which pockets of the active site will be occupied, contributing to their binding affinity and selectivity for the target. In this review, the ligand–protein co-crystal structures of c-Src kinase in complex with small-molecule inhibitors discovered in the past two decades are described, highlighting the crucial interactions of these compounds with the different portions of the protein binding site, thus providing a deeper understanding of the fundamental elements to be considered for the design of new and potent c-Src kinase inhibitors.

## 2. X-ray Structure Analyses

### 2.1. X-ray Structure of c-Src with Active and Inactive Kinase Conformation

**Figure 1:**
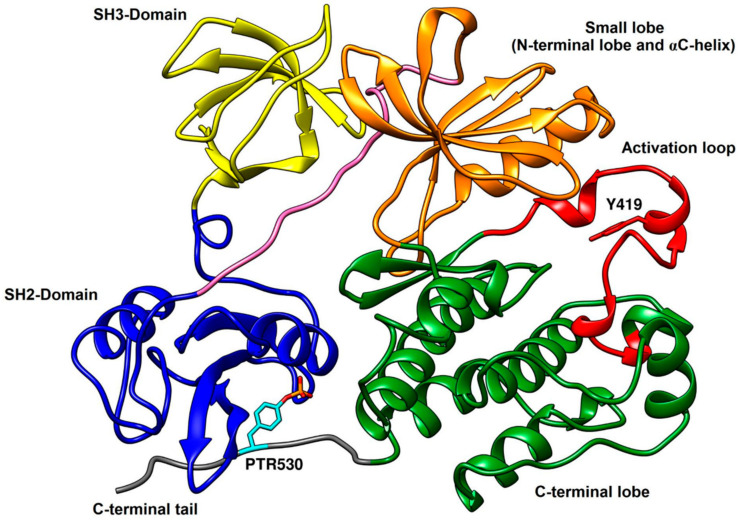
X-ray structure of c-Src in inactive conformation. The different domains are shown: SH1 domain comprising of small lobe (orange), activation loop (red), C-terminal lobe (green); SH2 domain (blue); SH3 domain (yellow); linker (pink); C-terminal tail (grey). Two phosphorylation sites Y419 and Y530 are labelled.

Xu and coworkers revealed in 1999 the X-ray structure of the full-length human c-Src kinase in inactive conformation, describing its autoinhibitory mechanism (Figure 1, PDB ID: 2SRC) [19]. The full-length c-Src is characterized by a multidomain architecture including: (a) a Src homology 1 (SH1) domain possessing tyrosine kinase activity, consisting of an N-terminal lobe and a C-terminal lobe; (b) a Src homology 2 (SH2) domain that binds to phosphotyrosine motifs; (c) a Src homology 3 (SH3) domain that typically binds to hydrophobic PxxP motifs; (d) a Src homology 4 (SH4) domain, which is a 15-amino acid peptide exposed to myristoylation, through which the kinase binds to the inner surface of plasma membrane; (e) a short C-terminal tail [20]. c-Src usually exists in a compact assembled inhibited conformation due to the phosphorylation of Y530, located in the C-terminal tail. The inactive conformation is maintained as a result of interdomain interactions between SH2 and SH3 domains, and this autoinhibitory state is defined as “assembled regulatory domain” conformation [21]. Dephosphorylation of Y530 and release of interdomain SH2–SH3 interactions weaken the assembled inactive conformation of c-Src, leading the kinase towards its catalytic conformation. However, phosphorylation of Y419 in the activation loop is essential to reach full catalytic potential. As soon as Y530 is dephosphorylated, subsequent phosphorylation occurs at Y419 via autophosphorylation that allows individual domains to retain their full-length architecture and activate the kinase [22]. Various small molecule peptidomimetics have been designed and tested to target SH2–SH3 domains so that the assembled inactive conformation of the kinase could be maintained for a prolonged catalytic inhibition [23].

**Figure 2:**
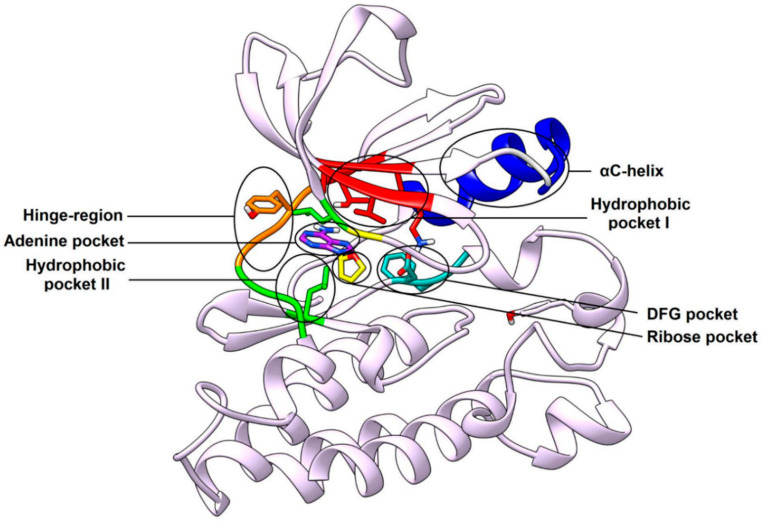
X-ray complex of inactive c-Src kinase showing the catalytic domain between N-terminal and C-terminal lobes, highlighted by different binding pockets. The DFG motif and the αC-helix are highlighted in cyan and blue, respectively.

The SH1 domain, which is also known as catalytic domain, demonstrates a typical bilobed structure comprising of N-terminal and C-terminal lobes, separated by a cleft where the catalytic activity of the kinase is accomplished with the transfer of a phosphate group from ATP to the substrate. The N-terminal lobe contains a highly conserved hinge-region, where ATP binds through H-bond interactions with M344 and E342. Physiologically, ATP binds to the cleft between the two lobes, with the phosphate group to be transferred to the substrate that is mainly bound to the activation loop of the C-terminal lobe [24,25,26]. The overall site where the whole ATP molecule binds is called ATP-binding site, which is composed of adenine pocket, adjacent to the hinge-region, and ribose pocket. The different binding pockets within the active site of c-Src kinase are depicted in Figure 2. The ribose pocket is lined by L276 and G277 of the N-terminal lobe and S348 of the C-terminal lobe. A deep hydrophobic pocket adjacent to hinge region and adenine pocket, which is surrounded by T341, K298, A296 and I339 of the N-terminal lobe, is denoted as hydrophobic pocket I. The position of the αC-helix belonging to the N-terminal lobe is an important determinant in kinase conformational activity. Some c-Src inhibitors, which bind to the inactive kinase conformation, usually occupy hydrophobic pocket I with large hydrophobic groups and displace the αC-helix outwards, causing the disruption of a salt bridge interaction between K298 of hydrophobic pocket I and E313 of the αC-helix (*vide infra*) [27]. T341, located at the entrance of hydrophobic pocket I, also called as gatekeeper residue, plays a crucial role in the selectivity of c-Src kinase inhibitors [28]. The activation loop of the C-terminal lobe is a major recognition site for enzyme substrates and another important determinant whose conformational changes are crucial for the activity of the kinase. The DFG motif of the activation loop, which is constituted by D407, F408 and G409 amino acid residues, undergoes significant conformational changes. Particularly, the outward flipping of the DFG lead to the formation of an additional hydrophobic pocket (often called DFG pocket) that would be otherwise occupied by F408 (*vide infra*). Another hydrophobic region, found in the cleft between the C-terminal lobe and the ribose pocket, is called hydrophobic pocket II, which is usually delimited by L276 and V284 of the N-terminal lobe and S348, G347, L396 of C-terminal lobe. Hydrophobic pocket II is occupied by the long tail of various ATP-competitive inhibitors, which further extend towards the solvent accessible area [28,29,30]. The design and development of potent inhibitors has focused mainly on exploiting the catalytic domain of c-Src kinase. The large cleft between the N-terminal and C-terminal lobes of the catalytic domain, including ATP-binding site (adenine and ribose pocket), hydrophobic pocket I, II and DFG pocket, constitute the binding site for the structure-based design of novel ligands.

### 2.2. X-ray Structure of c-Src Kinase in Complex with Potential Inhibitors

The availability of the X-ray structure of c-Src provided a great tool for the design and development of novel kinase inhibitors. Tremendous efforts were made in the past two decades to develop small-molecule inhibitors that could exploit the different pockets within the binding site of the enzyme. The foremost attempts were focused on the development of ATP-mimetics, which occupied adenine binding site and ribose pocket, and were able to reproduce the fundamental interactions with the target. Many ATP-mimetics have also shown to extend into hydrophobic pocket I and II, which contributed to the binding affinity for the kinase. These inhibitors belong to multiple classes of compounds (e.g., purine, pyrrolopyrimidine, pyrazolopyrimidne, naphthyridone, quinoline-based inhibitors and others) and are categorized as type-I kinase inhibitors [31]. Type-I inhibitors bind to the active kinase conformation and several X-ray structures of c-Src in complex with these ligands have been solved to date. A different category of c-Src kinase inhibitors is represented by type-II inhibitors, which bind to the inactive kinase conformation [32]. Two distinct elements characterize the inactive/active state of c-Src at the level of the binding site: DFG motif and αC-helix (colored in cyan and blue, respectively, in Figure 2) conformations. In the active kinase conformation, the conserved residue D407 of the DFG motif is oriented towards the catalytic site, since it should be able to interact with ATP and the Mg^2+^ ion to allow the catalytic reaction, while the side chain of F408 is oriented towards the inner part of the catalytic domain, repaired from the solvent. This conformation of the DFG motif is termed as DFG-in conformation. In the inactive state of the kinase, the DFG motif often undergoes a considerable rearrangement called DFG-out conformation, in which D407 moves towards the solvent, away from the binding pocket, while F408 completely flips around its backbone and places its side chain within the ATP binding site. This movement determines the opening of the so called DFG-pocket, an allosteric pocket adjacent to hydrophobic pocket I, which is instead occupied by F408 in the DFG-in conformation. The inhibitors that bind to the DFG-out conformation of the kinase and are able to interact with the DFG-pocket are called DFG-out type-II inhibitors. Similarly, the αC-helix of the N-terminal lobe can be subjected to an important conformational change in the inactive kinase state, in which a key salt bridge between the catalytic K298 and E313 is disrupted due to the displacement (and/or outward rotation) of the αC-helix. This particular conformation of the kinase is termed as αC-helix out (CHO) conformation, [27,33] which is often seen in the presence of inhibitors with large groups deeply accommodated into hydrophobic pocket I. The inhibitors which bind to the inactive kinase and stabilize its CHO conformation are called CHO type-II inhibitors. In contrast to these ligands, type-I inhibitors bind to the DFG-in and αC-helix-in conformation, typical of the active kinase state. Type-II inhibitors represent a class of ligands with a higher potential in terms of affinity and selectivity [34] towards the target kinase, due to an extended exploration of hydrophobic pocket I and, in particular, to the interactions with the DFG pocket, which is constituted by less conserved amino acids. Furthermore, the gatekeeper residue of c-Src kinase (T341), which is located at the entrance of hydrophobic pocket I, is often mutated to larger residues like methionine or leucine, resulting in steric clashes with the central core of type-I and type-II inhibitors, thus leading to drug-resistance. The nanomolar potent type-I inhibitor dasatinib offers a clear example of drug resistance, since it shows a complete loss of inhibition potency against the mutant T341M c-Src [35,36]. Some inhibitors of c-Src, designed to circumvent the mutation at the gatekeeper residue, only bind to the allosteric site of the catalytic domain and bypass the ATP-binding site. These inhibitors are known as allosteric type-III inhibitors, which avoid potential steric clashes with larger mutated gatekeeper residues and are endowed with great selectivity in kinase inhibition [37]. However, type-III inhibitors have not been largely explored like type-I and type-II inhibitors, because of their micromolar potency in kinase assays, whereas type-I and type-II inhibitors have often demonstrated nanomolar or sometimes picomolar potency against c-Src. A completely different category of c-Src kinase inhibitors involve SH2-SH3 domain peptidomimetics, where the intermolecular interactions of small peptide fragments (e.g., phosphotyrosine) with SH2/SH3 domain stabilizes the kinase in the assembled inactive conformation [23]. Drug design and discovery programs of kinase inhibitors are predominantly focused on exploitation of type-I, type-II inhibitors and their hybrids. Over the past two decades, various type-I and type-II inhibitors have been co-crystallized with human and chicken c-Src kinase. Chicken c-Src kinases share a very high sequence identity (94.2%) with human c-Src. More specifically, the binding site residues of chicken c-Src share about 99% identity in sequence with the binding site of human c-Src. X-ray structures of both human and chicken c-Src kinase in complex with type-I and type-II inhibitors, as well as few type-III allosteric ligands, have been covered in the present review to discuss the main ligand–protein interactions (Tables S1–S4). For clarity, the residue numbering of human c-Src has been used in the description of all co-crystal structures and related figures.

### 2.3. Type-I Inhibitors

The development of type-I inhibitors has led to successful results, since some outstanding candidates from type-I category were advanced to clinical trials and few of them, such as dasatinib, bosutinib and ruxolitinib, have been accepted as actual drugs. These compounds and other type-I inhibitors have been co-crystallized with c-Src (Table S2) to understand the structural bases of their affinity for the target.

**Figure 3:**
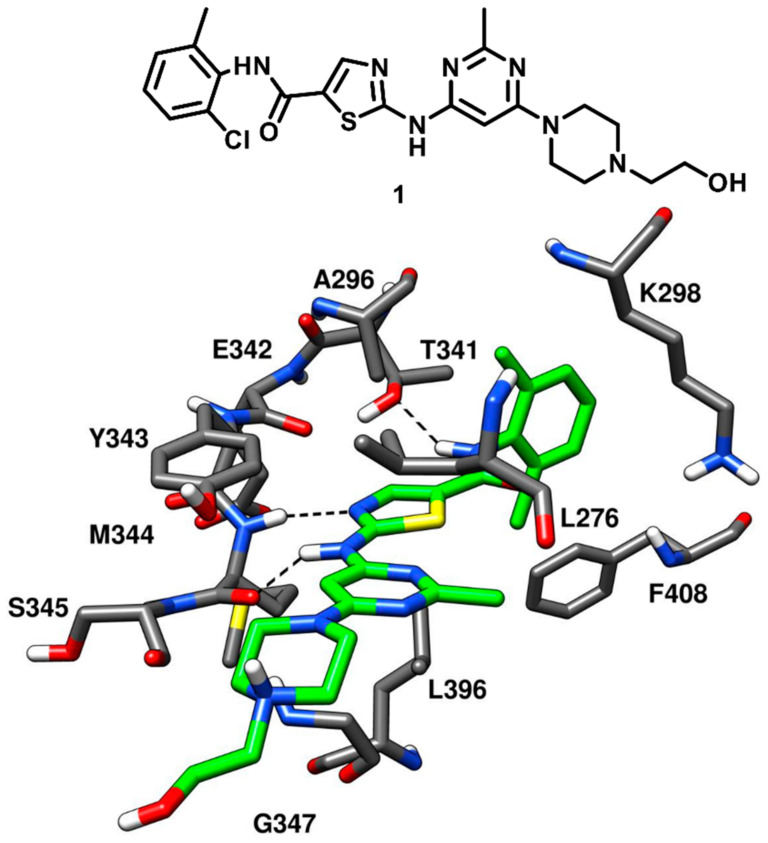
2D structure of **1** and X-ray structure of c-Src kinase in complex with **1** (PDB ID: 3G5D). Active site residues of c-Src kinase and the enzyme–inhibitor H-bond interactions are shown.

The first attempt to develop selective type-I inhibitors, which could competitively occupy the ATP-binding site of c-Src kinase was made by Lombardo and coworkers in 2004 [38]. The Lck kinase activity of 2-acylamino-5-carboxamidothiazoles had previously been reported by the same research group [16]. Based on the considerable results on Lck kinase activity, carboxamidothiazoles were rationally designed and substituted 2-(aminopyridyl) and 2-(aminopyrimidinyl) thiazole-5-carboxamides were synthesized and tested for c-Src inhibition properties. The first compound of the series, which is a 2-aminopyrimidine derivative, impressively displayed biochemical potency with a *K*_i_ of 96 pM for c-Src and an IC_50_ below 1.0 nM for Bcr-Abl kinase [39]. In general, both pyridine-based and pyrimidine-based analogues of the series demonstrated nanomolar to sub-nanomolar inhibitory potency against c-Src and Bcr-Abl kinase. The two 2-amino-thiazole pyrimidine derivatives with hydroxymethyl piperidine and hydroxyethyl piperazine tail at the fourth position of the pyrimidine ring were found to show the best balance in terms of broad spectrum antiproliferative activity against K562, PC3, MDA-MB-231 human breast tumor and WiDr human colon tumor cell lines. Both derivatives showed low-to-mid nanomolar inhibition potency against all cell lines and one of the two derivatives (compound **1**) was subjected to in vivo pharmacological evaluation through K562 xenograft mouse model. Compound **1** (BMS-354825, later known as dasatinib) which demonstrated an impressive IC_50_ of 0.50 nM for c-Src kinase, was co-crystallized with Abl kinase, revealing the key ligand–protein interactions with the enzyme. Compound **1** was expected to show a binding mode into c-Src comparable to that observed into the X-ray complex with Abl kinase. This was confirmed in 2009, when Getlik and coworkers solved the X-ray structure of active c-Src kinase in complex with compound **1** (PDB ID: 3G5D, Figure 3). Three H-bond interactions were formed by the ligand in the ATP-binding site of c-Src kinase. In particular, a pair of hydrogen bonds was observed in the highly conserved hinge region: a first one between the central NH group of compound **1** and the backbone oxygen of M344, and a second one between the nitrogen of the ligand thiazole ring and the backbone nitrogen of M344. Another important H-bond was formed between the gatekeeper residue T341 and the amide nitrogen of the inhibitor. As a type-I inhibitor, the ligand was bound to the active DFG-in conformation of the kinase with the 2-chloro-6-methylbenzamide moiety extending into hydrophobic pocket I, lined by the hydrophobic portions of T341 and K298. The orientation of the 2-chloro-6-methylbenzamide moiety provided important data for the structure-based design of new type-II inhibitors, which could bind to the DFG-out conformation and protrude deeper into hydrophobic pocket I (*vide infra*). The substituted pyrimidine moiety of compound **1** was located within hydrophobic pocket II, establishing van der Waals contacts with the sidechains of L276, G347 and L396 that account for the binding affinity towards the kinase. The hydroxyethylpiperazine tail of the inhibitor did not make any key contact with the protein residues and was mainly exposed to the solvent. Also, the ribose binding pocket of the catalytic domain was mostly unoccupied [38]. Compound **1**, was orally active in a K562 xenograft model of chronic myelogenous leukemia with high safety margin, later promoted into clinical trials due to its outstanding pharmacokinetic and in vivo profile. Today, compound **1** is an oral drug known as dasatinib and employed in a targeted therapy used for the treatment of chronic myelogenous leukemia (CML) and acute lymphoblastic leukemia (ALL) [40].

Significant efforts were made to exploit new heterocyclic scaffolds once the first aminothiazole-pyrimidine inhibitors of c-Src kinase with subnanomolar affinity were discovered. Cowan-Jacob and coworkers in 2004 employed a well-known antileukemia drug imatinib (STI571; Gleevec), with strong affinity for c-Abl but low inhibitory potency against c-Src kinase, as a starting point to develop new c-Src inhibitors [41]. During the structural optimization of imatinib, it was found that the incorporation of a methyl group in the lead compound from which imatinib was derived, reduced its inhibitory activity against c-Src kinase. The loss of activity could be probably due to the steric clash with the hinge-region produced by the methyl group on the central scaffold of the compound. In addition, imatinib showed high affinity for a specific inactive conformation of c-Abl but demonstrated very low affinity for the active conformation of c-Src kinase. Later in 2007, Seeliger and coworkers solved the X-ray structure of c-Src kinase in complex with imatinib [42] (compound **19**, discussed in the next section) to identify the binding mode of the ligand into c-Src and rationalize its weak inhibitory potency against c-Src.

**Figure 4:**
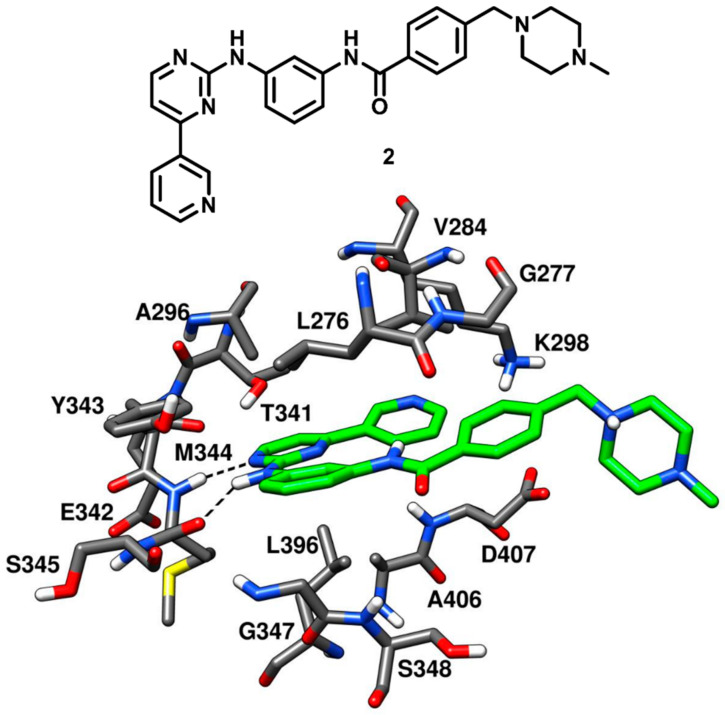
2D structure of **2** and X-ray structure of c-Src kinase in complex with **2** (PDB ID: 1Y57). Active site residues of c-Src kinase and the enzyme–inhibitor H-bond interactions are shown.

The des-methyl analog of imatinib (compound **2**) was tested against c-Src kinase, revealing an IC_50_ value of 1.6 nM in kinase inhibition assays. Compound **2** was co-crystallized with c-Src (PDB ID: 1Y57, Figure 4) that showed a new and partially assembled state in which the kinase domain adopted an active conformation. The X-ray structure was aimed to be utilized for the structure-based design of new c-Src inhibitors. Two of the four aromatic rings of the ligand, i.e., the terminal pyridine ring and the pyrimidinyl moiety, occupied the adenine binding site and were almost coplanar. Compound **2** formed two H-bonds with the backbone of M344 within the hinge-region. One H-bond was formed between the anilino-group of the inhibitor and the backbone oxygen of M344, while the other H-bond was observed between a pyrimidine nitrogen of compound **2** and the backbone nitrogen of M344. This X-ray structure confirmed that the presence of the methyl group in the inhibitor (like for imatinib) would have weakened these H-bonds due to the steric clashes with M344. The pyridine ring of the inhibitor established van der Waals contacts with V284, while the pyrimidine scaffold formed hydrophobic contacts with A296 and L396. The central phenyl moiety of the inhibitor laid on hydrophobic pocket II and established van der Waals interactions with L276 and G347. The phenyl ring of the lateral tail was located near G277 in the ribose pocket, while the terminal piperazine ring was largely exposed to the solvent [43].

**Figure 5:**
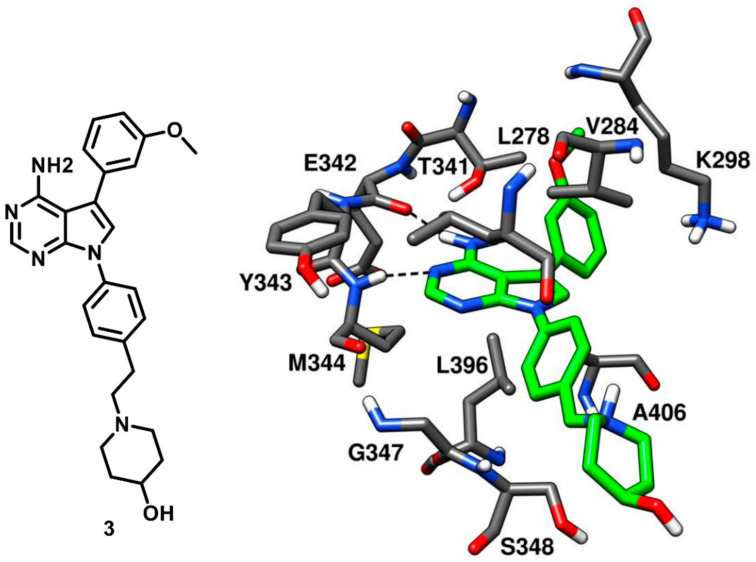
2D structure of **3** and X-ray structure of c-Src kinase in complex with **3** (PDB ID: 1YOL). Active site residues of c-Src kinase and the enzyme–inhibitor H-bond interactions are shown.

With the ongoing efforts in the development of c-Src kinase inhibitors, Breitenlechneret and coworkers in 2005 crystallized c-Src in active conformation and revealed how the kinase domain, in absence of the rigidifying interdomain interactions of the inactive state, adopts a more open and flexible conformation. The inhibitor CGP77675 (compound **3**) was co-crystallized with c-Src kinase (PDB ID: 1YOL, Figure 5) [20]. Compound 3 is a pyrrolopyrimidine derivative developed for the treatment of osteoporosis, with IC_50_ values for c-Src inhibition between 5 and 20 nM, depending on the substrate used [44]. The pyrrolopyrimidine moiety bound to the adenine pocket, analogously to the adenine group of ATP, and formed two H-bonds with the hinge region. One H-bond was formed between the pyrimidine nitrogen and the backbone nitrogen of M344 while the other H-bond was formed between the 4-amino group of the pyrrolopyrimidine ring and the backbone oxygen of E342. The inhibitor showed an additional H-bond between the 3-methoxy phenyl ring of the inhibitor and the hydroxyl group of the gatekeeper residue T341, which is believed to contribute to the selectivity for the kinase. Extensive van der Waals contacts with T341 and K298 were also formed by the methoxyphenyl substituent of the ligand, located into hydrophobic pocket I. The second phenyl ring occupied the ribose pocket and was partially exposed to the solvent, forming hydrophobic interactions with V284 and L396. The 4-hydroxypiperidine moiety was distant from the adenine binding pocket and mainly solvent exposed, similarly to the polar tail of compound **2** (Figure 4).

**Figure 6:**
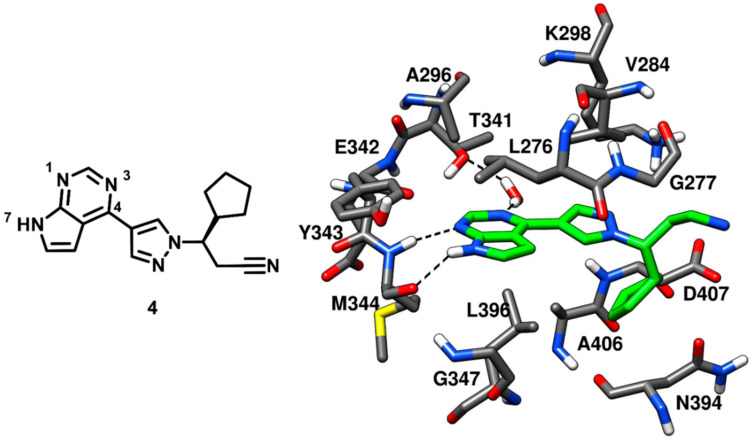
2D structure of **4** and X-ray structure of c-Src kinase in complex with **4** (PDB ID: 4U5J). Active site residues of c-Src kinase and the enzyme–inhibitor H-bond interactions are shown.

Ruxolitinib is an established anticancer drug, a potent janus kinase inhibitor approved for the treatment of myeloproliferative neoplasms. Ruxolitinib, which is a pyrrolopyrimidine inhibitor, demonstrated IC_50_ values of 3.3 nM and 2.8 nM against JAK1 and JAK2, respectively, but a weak inhibitory potency against c-Src kinase (with an IC_50_ value of 2.8 µM) [45,46]. Although the inhibitor was weakly potent against c-Src, no structural information about the bioactive conformation of ruxolitinib in respect to any kinase, was available. This led Duan and coworkers, in 2014, to further extend the investigation on the pyrrolopyrimidine scaffolds. Ruxolitinib (compound **4**) was co-crystallized with c-Src kinase (PDB ID: 4U5J, Figure 6) to identify its binding mode. The pyrrolopyrimidine ring was placed into the highly conserved ATP binding pocket and displayed two H-bonds within the hinge-region; one H-bond between N-1 and the backbone nitrogen of M344 and another H-bond between N-7 and the backbone oxygen of M344. Moreover, the N-3 of the pyrrolopyrimidine ring formed a water-mediated H-bond with the gatekeeper residue T341. The pyrrolopyrimidine central core demonstrated van der Waals contacts with L276 and A296 of the N-terminal lobe and L396 of the C-terminal lobe. The inhibitor was not found in the vicinity of the ribose pocket, which was left unoccupied. However, the cyclopentyl ring of the inhibitor was lined by N394 and D407, forming van der Waals contacts with these residues, while the pyrazole ring of compound **4** established additional van der Waals interactions with V284 of the N-terminal lobe. Unlike compound **3**, compound **4** did not present any groups that could occupy hydrophobic pocket I; thus suggesting that modifications of ruxolitinib aimed at occupying this pocket could lead to the development of more potent and promising c-Src inhibitors [47].

**Figure 7:**
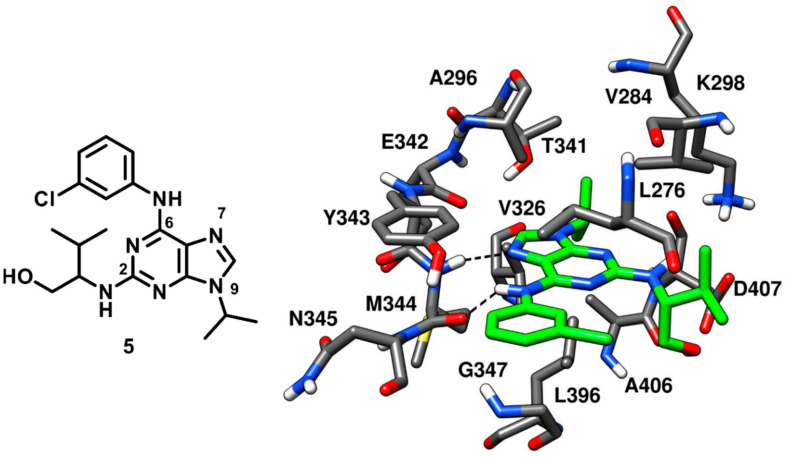
2D structure of **5** and X-ray structure of c-Src kinase in complex with **5** (PDB ID: 1YOM). Active site residues of c-Src kinase and the enzyme–inhibitor H-bond interactions are shown.

A tri-substituted purine-based inhibitor was investigated by the research group that already identified the pyrrolopyrimidine inhibitor **3**. Purvalanol A, reported to cause an arrest of G1 and G2 phase in cancer cell cycle progression in vitro, is a known low nanomolar inhibitor of cyclin-dependent protein kinases (CDKs) that also shows submicromolar affinity (with an IC_50_ of 0.24 µM) for c-Src kinase [48]. Purvalanol A (compound **5**) was co-crystallized with c-Src (Figure 7, PDB ID: 1YOM) and bound to the active kinase conformation. The inhibitor occupied the ATP-binding site of c-Src and established two H-bonds in the hinge-region; one H-bond between N-7 of the purine central core and the backbone nitrogen of M344, while the other H-bond between its 6-anilino group and the backbone oxygen of M344. The central purine ring of the molecule displayed van der Waals contacts with A296 and L396, while the N-9 isopropyl group of the inhibitor was found to interact with V326 of the C-terminal lobe, establishing additional hydrophobic contacts also with L396. The 3-chlorophenyl ring of compound **5** formed lipophilic interactions with L276 and G347 within hydrophobic pocket II, while the 3-methylbutanol chain occupied the ribose pocket of the binding site and showed van der Waals contacts with V284 of the N-terminal lobe [20]. The low nanomolar activity of compound **5** and its crystallographic structure served as a template for the structure-based design of more potent purine-based analogues which are discussed below.

**Figure 8:**
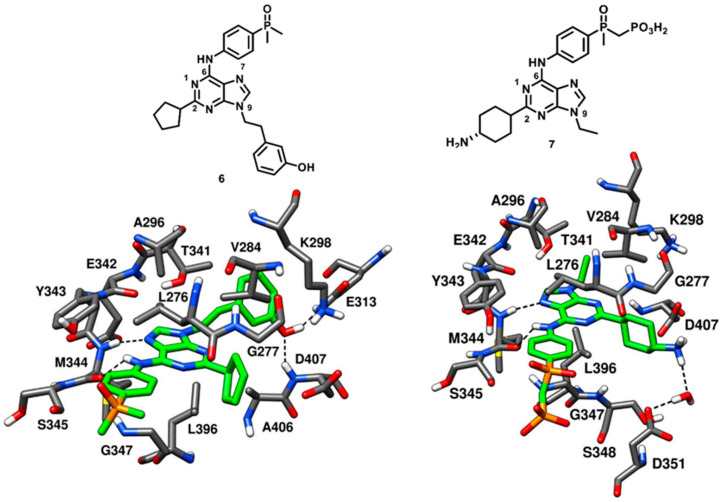
2D structures of **6** and **7**. X-ray structures of c-Src kinase in complex with **6** (PDB ID: 2BDJ) and **7** (PDB ID: 2BDF). Active site residues of c-Src kinase and the enzyme–inhibitor H-bond interactions are shown.

Dalgarno and coworkers in 2006 adopted an approach for exploiting rationally designed compound libraries of 2,6,9-trisubstituted purines to achieve selective inhibition of c-Src kinase. The compound optimization was aided by molecular docking on c-Src X-ray structure, followed by synthesis and kinase inhibition assays. Purvalanol A (compound **5**) was used as starting point in the chemical optimization focused on synthetic modifications at the 2-, 6- and 9-positions of the purine nucleus with the aim of obtaining new SAR data [49]. Two lead compounds AP23464 (compound **6**) and AP23451 (compound **7**) were identified. Compound **6** displayed an exceptional and enhanced c-Src inhibitory potency, with an IC_50_ of 0.45 nM, and a weak CDK2 inhibitory potency, with an IC_50_ of 20.9 µM, thus demonstrating more than 46000-fold selectivity for c-Src kinase over CDK2. Compound **7** was designed with the aim of inhibiting c-Src kinase-dependent bone resorption [50]. For this purpose, a bone-targeting phosphonomethylphosphinyl (PCP) chemical moiety was introduced into compound **7** at the para-position of its aniline ring. The incorporation of the PCP moiety favored the localization of the compound into to the bone, although the cellular permeability of the inhibitor was limited due to the presence of a charged moiety [51]. The inhibitor showed a remarkable inhibitory activity against c-Src (IC_50_ of 0.067 µM) and displayed a 30-fold selectivity over CDK2 (IC_50_ = 2.01 µM). Both compounds **6** and **7** were co-crystallized with c-Src kinase (PDB IDs: 2BDJ and 2BDF, respectively, Figure 8). The inhibitors well occupied the ligand binding site between the N-terminal and C-terminal lobes, interacting with the kinase in active conformation. Though both inhibitors are ATP site binders, the orientation of the tri-substituted purine ring significantly differed from ATP, as observed for Purvalanol A. Both inhibitors established H-bonds with the backbone nitrogen and oxygen of M344 through their N-7 and aniline nitrogen, respectively. The purine nucleus of both inhibitors was sandwiched between A296 and L396, forming van der Waals interactions with these residues. The C-2 substituents of compounds **6** and **7**, corresponding to a 4-amino cyclohexyl and a cyclopentyl moiety, respectively, were located at the ribose pocket, contributing to fill the wide cleft between the N-terminal and C-terminal lobes of the kinase and showing van der Waals contacts predominantly with V284 and L396. The introduction of the 3-hydroxyphenethyl substituent into compound **6** allowed the formation of interactions with the binding site, which were considered critical for its picomolar affinity. This unique structural component of compound **6** penetrated deeply into hydrophobic pocket I and formed H-bonds with the carboxylic group of E313 and the backbone nitrogen of D407. Unlike compound **6**, compound **7** bore a shorter N-9 ethyl substituent which was projected between A296 and T341, and only showed van der Waals contacts with the two residues. The shorter N-9 substituent of compound **7** lacked the ability to deeply occupy hydrophobic pocket I and thus could not form H-bonds with E313 and D407. Nevertheless, the terminal amino group of the cyclohexyl moiety of compound **7** showed a water-mediated H-bond interaction with the carboxylic group of D351. Both compounds **6** and **7** were characterized by the presence of an aniline ring at C-6 position, endowed with a para-substituted phosphine oxide and a phosphonate group, respectively. The anilino-moiety of both inhibitors established van der Waals contacts mainly with L276 and G347 in the hydrophobic pocket II. However, the terminal dimethyl phosphine oxide (compound **6**) and phosphonate group (compound **7**) did not make any contact with the catalytic domain and were largely exposed to solvent [50]. The identification of compounds **6** and **7** was regarded as a breakthrough in the development of c-Src kinase inhibitors, especially in relation to compound **6**, which is one of the most potent reversible inhibitors reported so far.

**Figure 9:**
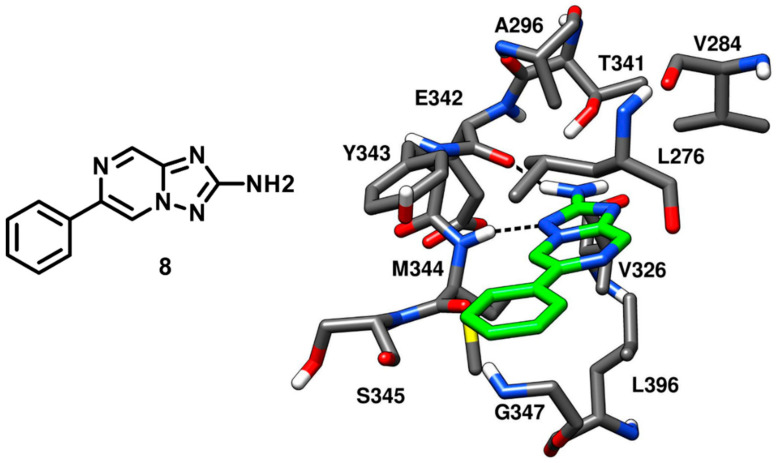
2D structure of **8** and X-ray structure of c-Src kinase in complex with **8** (PDB ID: 4FIC). Active site residues of c-Src kinase and the enzyme–inhibitor H-bond interactions are shown.

Robert and coworkers in 2013 performed an extensive structure-based de novo design of protein kinase inhibitors. A virtual screening (VS) protocol was implemented with the following steps: (a) core fragment extraction out of commercially available compounds, (b) selection of candidate core fragments by filtering out unwanted functionalities, (c) 3D-pharmacophore search to screen fragments with hinge-region binding moieties, (d) docking of core fragments into X-ray structures of several different kinases and (e) fragment expansion by synthesis. At the end of the VS study, six new compounds were obtained and tested against a panel of different kinases, showing c-Src inhibitory activity in the micromolar range. One of the best inhibitors of the series (compound **8**), containing a triazolopyrazine core, demonstrated an IC_50_ of 20 µM against c-Src and was co-crystallized with the kinase (PDB ID: 4FIC, Figure 9) to validate the proposed binding mode [52]. The 2-amino group of the bicyclic triazolopyrazine ring formed an H-bond with the backbone oxygen of E342, while the adjacent nitrogen of the triazole established another H-bond with the backbone nitrogen of M344. These two H-bond interactions well mimicked those displayed by ATP in the hinge-region. Moreover, the phenyl substituent of compound **8** formed extensive van der Waals contacts with L276 and G347 within hydrophobic pocket II that certainly contributed to its binding affinity for the target. However, compound **8** lacked a group that could occupy hydrophobic pocket I like other type-I inhibitors, which justified its micromolar potency. Although the inhibitor being weakly potent, it satisfied the Lipinski rule of 5 [53] and the triazolopyrazine ring was identified as a new scaffold for optimization studies where hydrophobic groups could be incorporated for the discovery of new c-Src inhibitors with improved selectivity and affinity.

**Figure 10:**
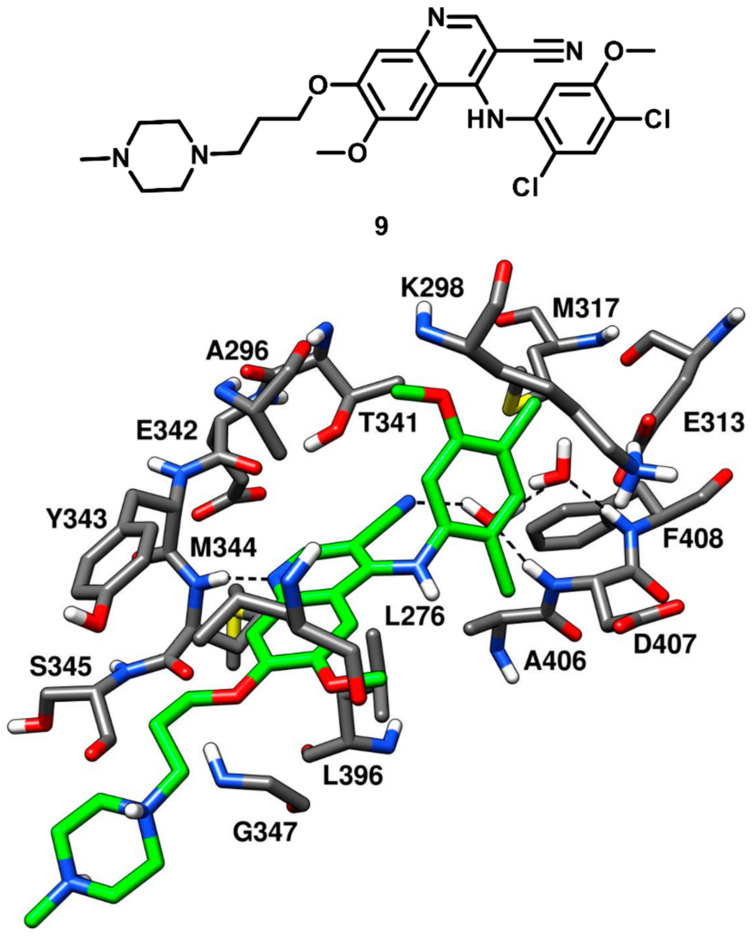
2D structure of **9** and X-ray structure of c-Src kinase in complex with **9** (PDB ID: 4MXO). Active site residues of c-Src kinase and the enzyme–inhibitor H-bond interactions are shown.

The benzopyridine inhibitor bosutinib (SKI-606) developed by Pfizer, was FDA-approved in 2017. Bosutinib is a Abl and c-Src tyrosine kinase inhibitor, used for the treatment of chronic myelogenous leukemia and marketed under the trade name Bosulif [54]. In a cell-free assay, bosutinib demonstrated an IC_50_ of 1.2 nM against c-Src kinase and was found to be selective for c-Src over non-Src family kinases. The compound potently inhibited Src-dependent cell proliferation in rat fibroblasts with an IC_50_ of 0.1 µM. Back in 2014, Levinson and Boxer co-crystallized bosutinib (compound **9**) with c-Src kinase (PDB ID: 4MXO, Figure 10) for getting deeper insights on its selectivity for c-Src over other kinases [55]. The same research group, in 2012, co-crystallized bosutinib with Abl kinase and revealed that the inhibitor bound to a DFG-out conformation of the kinase (PDB ID: 3UE4) [56]. Similar results were expected from the X-ray complex of c-Src, but the kinase demonstrated a DFG-in active conformation when bound to the inhibitor. The benzopyridine core of compound **9** was encircled by the side chains of A296 and L276 of the N-terminal lobe and L396 of the C-terminal lobe, thus forming hydrophobic interactions with these residues. A classical H-bond interaction was observed in the ATP binding site, between the pyridine nitrogen of compound **9** and the backbone nitrogen of M344. Additional van der Waals interactions were established by the propyloxy-linker of compound **9** with G347 and L276 in the hydrophobic pocket II, while the piperazine tail was typically exposed to the solvent, similarly to other inhibitors. The ribose pocket of the binding site remained unoccupied, while hydrophobic pocket I was occupied by the dichloromethoxyphenyl ring of the inhibitor. The gatekeeper residue T341 of c-Src formed van der Waals contacts with the 3-nitrile group and the dichloromethoxyphenyl ring of the ligand, which was also seen in bosutinib-Abl complex. Levinson and Boxer also reported that most of the type I inhibitors left a small cavity next to the gatekeeper residue, adjacent to hydrophobic pocket I, unoccupied. Within this cavity, two structural water molecules are often found, showing a network of H-bonds in which the bound inhibitor often participates. Interestingly, a pair of conserved water molecules mediating H-bond interactions between the ligand and the activation loop of the kinase was identified in the c-Src-bosutinib X-ray complex. In particular, the DFG-in active conformation of the kinase revealed that the backbone NH groups of both D407 and F408 of the DFG motif pointed towards the small cavity adjacent to hydrophobic pocket I, anchoring two ordered water molecules. The 3-nitrile group of the inhibitor was oriented towards the same cavity and formed an H-bond with one of the two water molecules in the binding site, thus participating in a water-mediated H-bond network with D407, F408 and E313. Bosutinib-Abl kinase complex demonstrated that the structured-water molecules were oriented differently in the cavity, since the kinase adopted a DGF-out conformation where F408 was flipped outwards. These water-mediated interactions highlighted a crucial element for the inhibitor’s ability to differentiate between different kinases (e.g., c-Src and Abl) and suggested that a greater attention to structured water molecules within the kinase active site is required for the design of selective inhibitors.

As discussed in the introduction, the gatekeeper residue in the ATP-binding site is supposed to play a crucial role in the inhibitors’ selectivity profile, being very prone to undergo mutation and replacement by bigger residues like methionine or isoleucine. In fact, patients undergoing anticancer therapies based on kinase inhibitors frequently develop clinical resistance mediated by mutations at this position [35,36]. For this reason, bosutinib was co-crystallized with T341M-M317L double mutant (PDB ID: 4MXZ) and A406T single mutant (PDB ID: 4MXX) c-Src with the aim of investigating the role of the water-mediated interactions for bosutinib’s activity in presence of clinically relevant mutated c-Src residues, with respect to wild type c-Src (Figure S1). Crystallographic observations from T341M/M317L c-Src structure highlighted a slight closure of the N-terminal and C-terminal lobes of the kinase domain, resulting in the rotation of the aniline ring of bosutinib towards the larger and hydrophobic gatekeeper residue. The 4-chloro group of the aniline ring hampered the disposition of the two structural water molecules within the pocket, which led to the loss of the water-mediated H-bonds with the nitrile group of bosutinib. In the A406T c-Src complex, the sidechain of T406 displaced the structural water molecules and consequently formed a direct H-bond with the nitrile group of bosutinib. The dissociation constants of bosutinib for wild-type c-Src and both mutant c-Src were measured using a fluorescence assay. The K_d_ value of bosutinib for wild type c-Src was found to be 0.73 nM whereas the K_d_ value obtained for T341M/M317L and A406T c-Src mutants were found to be 30-fold (21.2 nM) and 40-fold (29 nM) lower, respectively. These data confirmed that the mutation at the gatekeeper and A406 residues interferes with the water-mediated H-bond network of bosutinib and results in significant loss of binding affinities.

**Figure 11:**
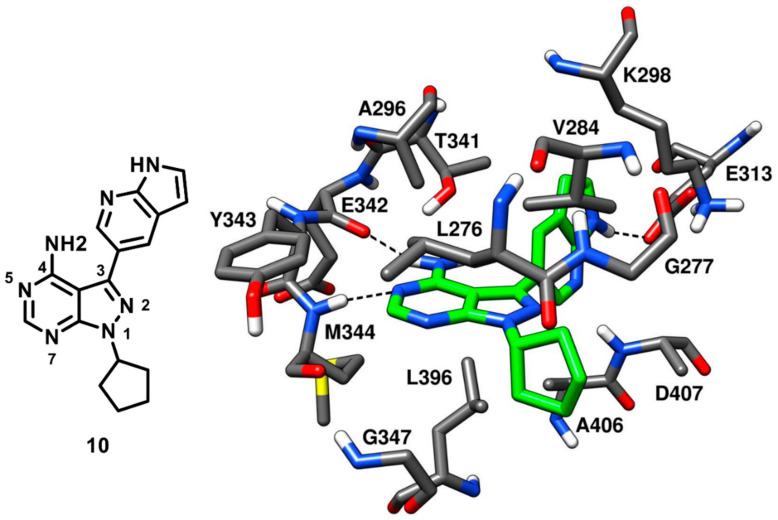
2D structure of **10** and X-ray structure of c-Src kinase in complex with **10** (PDB ID: 3EN4). Active site residues of c-Src kinase and the enzyme–inhibitor H-bond interactions are shown.

Apsel and coworkers in 2008 carried out an extensive research on the discovery of multitarget kinase inhibitors containing a pyrazolopyrimidine scaffold. An experimental screening of tyrosine kinase inhibitors for activity against the PI3-K p110α resulted in the identification of two pyrazolopyrimidines with nanomolar inhibitory potency that inspired the synthesis of more than 200 derivatives, which were tested against a panel of 14 tyrosine kinases and PI3-Ks. Four new pyrazolopyrimidine inhibitors that displayed interesting inhibition properties for c-Src kinase were identified from robust SAR studies. All four pyrazolopyrimidines were co-crystallized with c-Src [57], which further revealed that one ligand (compound **10**, Figure 11), which showed an IC_50_ of 14 nM against c-Src kinase, was a type-I inhibitor, while the other three were CHO type-II inhibitors (see next section for their description). The central pyrazolopyrimidine core of compound **10** occupied the adenine pocket of the ATP-binding site and formed two typical H-bonds in the hinge-region (PDB ID: 3EN4, Figure 11). The 4-amino group of the pyrazolopyrimidine ring established an H-bond with the backbone oxygen of E342 while the 5-N of pyrazolopyrimidine ring formed another H-bond with the backbone nitrogen of M344. The C-3 pyrrolopyridine substituent of the inhibitor was projected towards the gatekeeper residue into hydrophobic pocket I, showing hydrophobic interactions with T341 and K298 and a crucial H-bond with the carboxylic group of E313, which is usually implicated in the salt bridge with K298 and in maintaining the αC-helix-in active kinase conformation. This particular H-bond interaction was not observed for other members of the series and is believed to contribute to the nanomolar potency of the inhibitor. The solvent-exposed cyclopentyl substituent of the inhibitor was well accommodated into the ribose pocket of the binding site showing van der Waals contacts with V284. Despite its high inhibitory potency against c-Src, compound **10** was found to have poor selectivity, as it showed a broad-spectrum inhibition profile with nanomolar potency against other kinases, such as Ret (IC_50_ < 1 nM), VEGFR2 (IC_50_ = 12 nM) and Abl kinase (IC_50_ = 18 nM).

Recently, Tintori and coworkers have conducted a research focused on the development of novel c-Src/Abl inhibitors endowed with a pyrazolopyrimidine scaffold [58,59,60,61]. Several members of this series were found to induce apoptosis and to reduce cell proliferation in various solid tumor cell lines. In particular, compound **11a**, characterized by a C-6 methylthio group on the pyrazolopyrimidine scaffold (Figure S2), demonstrated a promising antiproliferative activity in SH-SY5Y cell cultures of human neuroblastoma [62]. Compound 11a displayed an appreciable selectivity for c-Src when tested against a panel of tyrosine kinases (EGFR, KDR, Flt-3, IGF-IR, Tek and c-Src) [63].

**Figure 12:**
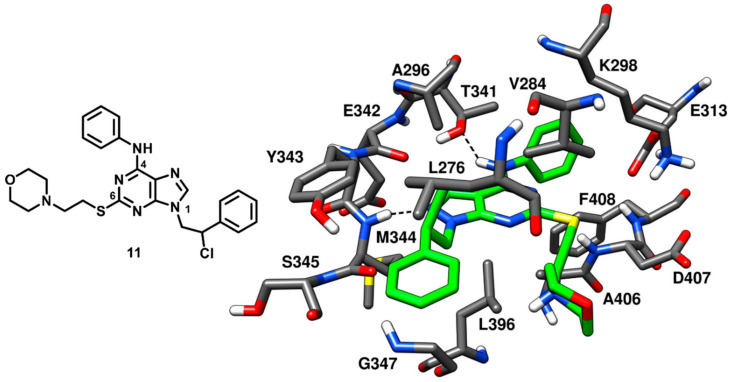
2D structure of **11** and X-ray structure of c-Src kinase in complex with **11** (PDB ID: 4O2P). Active site residues of c-Src kinase and the enzyme–inhibitor H-bond interactions are shown.

A small library of pyrazolopyrimidine analogues of compound **11a** was designed and synthesized, aimed at better exploring N-1, C-4 and C-6 positions of the central scaffold with SAR studies. Molecular modeling suggested that the C-6 substituent of the pyrazolopyrimidine analogue was oriented toward the solvent-exposed region of c-Src binding site and SAR studies highlighted that the introduction of polar groups in C-6 position would be suitable for enhancing the water solubility of the compounds. This study led to the identification of a compound **11** (Figure 12) that displayed a significantly improved profile in terms of both biological activity and ADME properties. Compound **11** was characterized by a high metabolic stability (95%), good water solubility (1.7 µg/mL) and a potent inhibitory activity against isolated c-Src (Ki = 0.21 µM). Compound **11** was co-crystallized with c-Src kinase (PDB ID: 4O2P, Figure 12) to get deeper structural insights on the binding mode of this series of compounds and employ the X-ray complex for the structure-based design of more potent analogues. The central pyrazolopyrimidine moiety of the compound exhibited hydrophobic contacts with V284, A296 and L396, while its N-2 nitrogen showed a typical interaction with the backbone nitrogen of M344 located in the hinge region. The amino group of the C-4 aniline substituent of compound **11** formed a second H-bond with the gatekeeper residue T341 and the aromatic ring was placed between T341 and K298 in the hydrophobic pocket I, forming van der Waals interactions with these residues. The flexible ethylthio linker at C-6 position of the scaffold allowed the disposition of the morpholine ring in the ribose pocket, while the terminal phenyl ring of the N1 substituent was sandwiched between L276 and G347, thus occupying hydrophobic pocket II. Finally, the chlorine atom of the ligand was found to be in close proximity with L276 and G347 of hydrophobic pocket II and displayed strong van der Waals contacts with these residues. Compound **11** presented a chiral center and the racemic mixture was used for the preparation of the X-ray crystal structure; notably only the (*R*)-enantiomer was able to bind the active conformation of the kinase, despite no differences in biological activity was observed for the two enantiomers [64].

**Figure 13:**
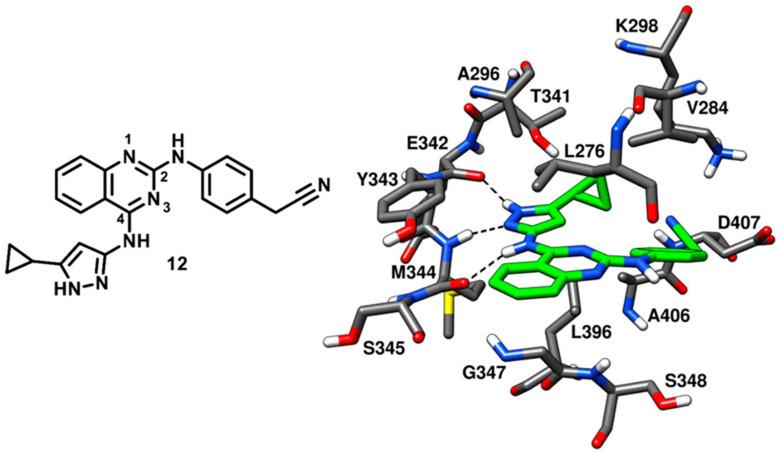
2D structure of **12** and X-ray structure of c-Src kinase in complex with **12** (PDB ID: 3F6X). Active site residues of c-Src kinase and the enzyme–inhibitor H-bond interactions are shown.

In 2008, Statsuk and coworkers focused on the discovery of multitarget kinase inhibitors and identified a moderately potent quinazoline-based inhibitor (**12**) of c-Src, which served as a template in the optimization studies for the development of potent quinazoline-based c-Src ligands in the following years. Compound **12** was screened against a panel of 359 wild type kinases and found to bind most of them. The inhibitor displayed 0.1% residual c-Src kinase activity, as compared to control, in the enzyme assays and emerged as a mid-micromolar potent inhibitor of c-Src (*Kd*~500 nM). Compound **12** was co-crystallized with c-Src (PDB ID: 3F6X, Figure 13), showing to act as a type-I inhibitor, interacting with the active kinase conformation [65]. The 4-amino pyrazole moiety of the inhibitor was accommodated into the adenine pocket and formed three H-bonds in the hinge-region of the binding site. In particular, the pyrazole ring displayed two H-bonds with the backbone oxygen of E342 and the backbone nitrogen of M344, while the 4-amino group showed an H-bond with the backbone oxygen of M344. Moreover, the 5-cyclopropyl group of the pyrazole ring laid in front of the gatekeeper residue, thus establishing van der Waals contacts with T341 and L396 of the C-terminal lobe. The central quinazoline core of compound **12** mainly showed van der Waals contacts with L276 and G347 in hydrophobic pocket II. The phenyl ring of the cyanobenzyl moiety occupied the ribose pocket and predominantly formed lipophilic interactions with V284, while the nitrile group showed van der Waals contacts with the side chains of D407 and K298. 

**Figure 14:**
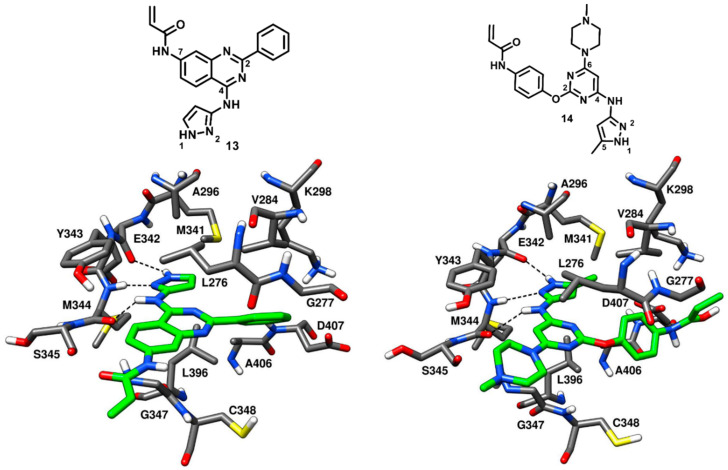
2D structures of **13** and **14**. X-ray structures of c-Src kinase in complex with **13** (PDB ID: 5D12) and **14** (PDB ID: 5D10). Active site residues of c-Src kinase and the enzyme–inhibitor H-bond interactions are shown.

Engel and coworkers in 2015 designed and synthesized a series of irreversible quinazoline and pyrimidine-based EGFR inhibitors. The best compounds of the series were co-crystallized with engineered c-Src (T341M/S348C), which is a validated model system for EGFR and drug-resistant EGFR (T790M) [36,66]. However, two compounds of the series (**13** and **14**, Figure 14) were found binding to c-Src in a reversible manner. From the activity assays, the quinazoline-based inhibitor **13** demonstrated moderate inhibition of wild-type c-Src, with an IC_50_ of 0.211 µM, but showed a 6- to 9-fold stronger potency against mutant c-Src, with IC_50_ values of 65 nM and 43 nM against T341M c-Src and T341M/S348C c-Src, respectively. Similarly, the pyrimidine-based inhibitor **14** displayed IC_50_ values of 0.218 µM, 0.107 µM and 72 nM against wild-type c-Src, T341M c-Src and T341M/S348C c-Src, respectively. 

In order to gain structural insights into their binding mode, both **13** and **14** were co-crystallized with T341M/S348C mutated c-Src (PDB ID: 5D12 and 5D10, respectively, Figure 14) [67]. The 4-amino pyrazole moiety of both inhibitors occupied the adenine pocket, as observed for compound **12**, and formed three H-bonds with the hinge-region residues. The 4-amino group of both **13** and **14** formed an H-bond with the backbone oxygen of M344, while the pyrazole ring established H-bonds with the backbone oxygen of E342 and the backbone nitrogen of M344. The orientation of the pyrazole ring of compound **13** was not affected by the mutated and larger gatekeeper residue M341; however, the presence of the 5-methyl group in the pyrazole ring of compound **14** displaced M341 away from the binding site due to steric repulsions and could be a reason of the slightly decreased potency of **14** with respect to **13**. The central quinazoline ring of **13** and the pyrimidine ring of **14** established van der Waals interactions with L276 and G347 in the hydrophobic pocket II. The Michael acceptor (acrylamide) in the 7-position of the quinazoline inhibitor **13** projected outward from the ATP binding pocket, into the solvent-exposed region, thus preventing the covalent interaction with C348, whereas the 2-phenyl ring of the quinazoline core laid near the ribose pocket, showing van der Waals contacts mainly with V284. The methylpiperazine tail of **14** was solvent exposed, acting as a solubilizing group, while the 2-phenoxy ring linked to the pyrimidine core showed hydrophobic contacts with V284 similarly to phenyl ring of **13**. The terminal acrylamide moiety of compound **14** was projected away from the binding site leaving no chance of covalent bonding with C348. The binding mode of compound **13** and **14** in complex with T341/S348C c-Src showed certain similarities to other reversible type-I inhibitors and due to the observed tolerance with larger mutated gatekeeper residues, the two compounds were further employed for the structure-based design of drug-resistant c-Src (T341M) inhibitors.

### 2.4. Type-II Inhibitors

Most type-I inhibitors are not able to fully occupy hydrophobic pocket I and to interact with the DFG pocket, leaving space within the kinase active site that could be targeted in order to improve their binding affinity. The design of type-II inhibitors has been mainly focused on extending the binding interactions of the ligands into these pockets. Successful strategies of type-II inhibitors design involved the structural optimization of known type-I inhibitors (e.g., design of type-II pyrazolopyrimidine inhibitors [68]) and a hybrid design approach (e.g., design of dasatinib–imatinib hybrid compounds [69]). Type II inhibitors bind inactive kinase conformations of c-Src and often show advanced pharmacological properties, such as slow dissociation rates, which increase drug-target residence time and result in significant enhancement in affinities over type I inhibitors. In the following paragraph, a description of the ligand–protein interactions of some important type-II inhibitors co-crystallized with c-Src kinase is reported (Table S3).

**Figure 15:**
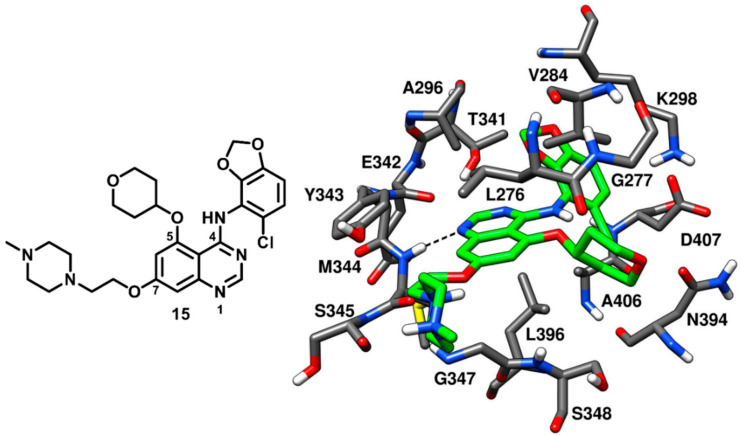
2D structure of **15** and X-ray structure of c-Src kinase in complex with **15** (PDB ID: 2H8H). Active site residues of c-Src kinase and the enzyme–inhibitor H-bond interactions are shown.

In 2004, Ple and coworkers reported a series of 2-chloro-5-methoxyanilinoquinazolines substituted at the C-6 and C-7 positions of the quinazoline ring that demonstrated good inhibition potency against c-Src [70]. However, molecular modeling studies suggested that the inhibitors were not able to occupy the ribose pocket of the kinase. Later on, the same research group investigated a new series of anilinoquinazolines substituted at the C-5 position of the quinazoline ring [71]. Docking results confirmed that the C-5 position allowed the substituents to access the ribose pocket in terms of shape complementarity. The design of C-5 substitued anilinoquinazolines was derived from the discovery of CDK1-2 (cyclin-dependent kinase) inhibitors and from docking studies suggesting that the ribose pocket could be occupied by a broad range of substituents, including straight or branched alkyl chains, phenyl rings and cyclic amines [72]. A total of 42 quinazoline compounds bearing either a 2-chloro-5-methoxyanilino or a chlorobenzodioxolylamino group were synthesized. An extensive SAR study was aimed at modifying the C-5 position of the quinazoline core to identify suitable substituents that could satisfactorily occupy the ribose pocket and improve the binding affinity for the target. Cyclic substituents, especially oxygen and nitrogen containing heterocycles, were found to be more favorable over acyclic and flexible substituents at C-5 position, leading to the identification of potent enzyme inhibitors. This class of compounds proved to be highly selective for c-Src over a variety of other kinases, such as CDK2, Aurora kinase, EGFR-TR, MEK and VEGFR-2. In particular, C-5 substituted anilinoquinazolines were reported to be 70-fold more potent towards c-Src over VEGFR-2 [70]. The best compound of the series, compound **15** (AZD0530, saracatinib, Figure 15), is a bicyclic [(chlorobenzodioxolyl)amino]-quinazoline with a tetrahydropyranyloxy group at the C-5 position, which demonstrated a remarkable IC_50_ of 2.7 nM in c-Src kinase inhibition. Compound **15** was co-crystallized with c-Src kinase (Figure 15, PDB ID: 2H8H) and showed binding to the inactive kinase conformation. The tetrahydropyran ring of the inhibitor occupied the ribose pocket, forming hydrophobic interactions with L276, V284 (N-terminal lobe) and L396 (C-terminal lobe). The binding of the inhibitor determined a slight distortion of the ATP pocket and allowed a little opening at the entrance of hydrophobic pocket I to accommodate the bulky benzodioxane ring. Due to the presence of the large benzodioxane group, the αC-helix of the catalytic domain was displaced outwards, causing the disruption of the salt bridge interaction between K298 and E313. The displacement of the αC-helix categorized compound **15** as a CHO (αC-helix-out) type-II inhibitor. The chlorine atom on the benzodioxane ring established lipophilic interactions with the methyl side chain of A406. Similarly to type-I inhibitors, compound **15** displayed a key H-bond interaction in the hinge-region of the ATP-binding site, since the N-1 of the quinazoline core formed an H-bond with the backbone nitrogen of M344. The piperazine tail of compound **15** laid predominantly on the solvent exposed region, whereas the ethyloxy linker established van der Waals contacts with L276 in hydrophobic pocket II. The discovery of type-II quinazoline inhibitors allowed a better exploration of hydrophobic pocket I and ribose pocket of the c-Src binding site, which could be utilized for the development of promising type-II analogues.

**Figure 16:**
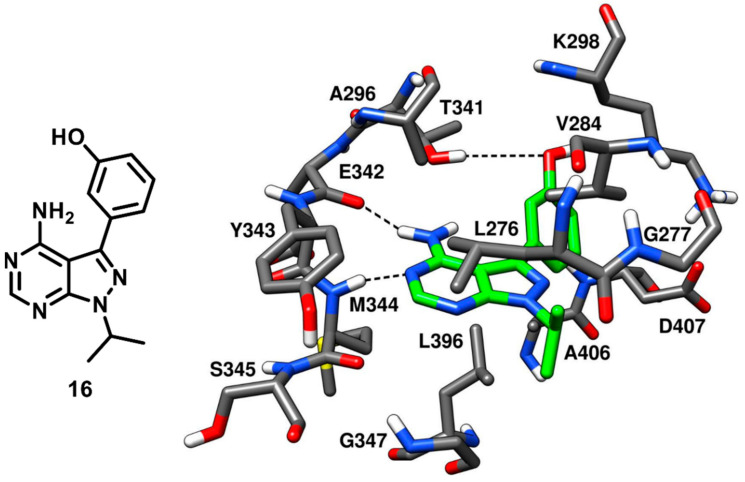
2D structure of **16** and X-ray structure of c-Src kinase in complex with **16** (PDB ID: 3EN7). Active site residues of c-Src kinase and the enzyme–inhibitor H-bond interactions are shown.

Compound **10** bearing a pyrazolopyrimidine scaffold, which was discussed earlier in type-I inhibitors’ section, was identified along with three more pyrazolopyrimidine inhibitors that were co-crystallized with c-Src. These ligands were able to bind the inactive kinase with CHO conformation, where the key salt bridge interaction between K298 and E313 was disrupted. Among the three inhibitors, compound **16** (PDB ID: 3EN7, Figure 16) was the most potent, with an IC_50_ of 15 nM, similarly to compound **10**, while compounds **17** and **18** (PDB IDs 3EN5 and 3EN6 respectively, Figure S3) showed IC_50_ values of 360 nM and 235 nM, respectively [57]. The central pyrazolopyrimidine core of all three inhibitors occupied the adenine pocket and showed two H-bond interactions with the hinge-region: as observed for compound **10**, the 4-amino group of the pyrazolopyrimidine ring established an H-bond with the backbone oxygen of E342 and the 5-N nitrogen formed the second H-bond with the backbone nitrogen of M344. The C-3 aryl substituents of these inhibitors (the phenol, dimethoxyphenyl and quinoline groups of **16**, **17** and **18**, respectively) extended beyond the gatekeeper residue and were accommodated into the hydrophobic pocket I. The solvent exposed N-1 cyclic and acyclic substituents (isopropyl for **16**, **18** and cyclobutyl for **17**) occupied the ribose pocket and established van der Waals interactions with V284, L276 and G277 of the N-terminal lobe. The key feature that differentiated compound **16** from the other two ligands is that **16** was able to form a third additional H-bond with the gatekeeper residue, which significantly improved its inhibition potency over compound **17** and **18**. Notably, the type-I analogue **10** that showed an IC_50_ for c-Src of 14 nM was also able to form a third H-bond in addition to the H-bond interactions with the hinge region.

**Figure 17:**
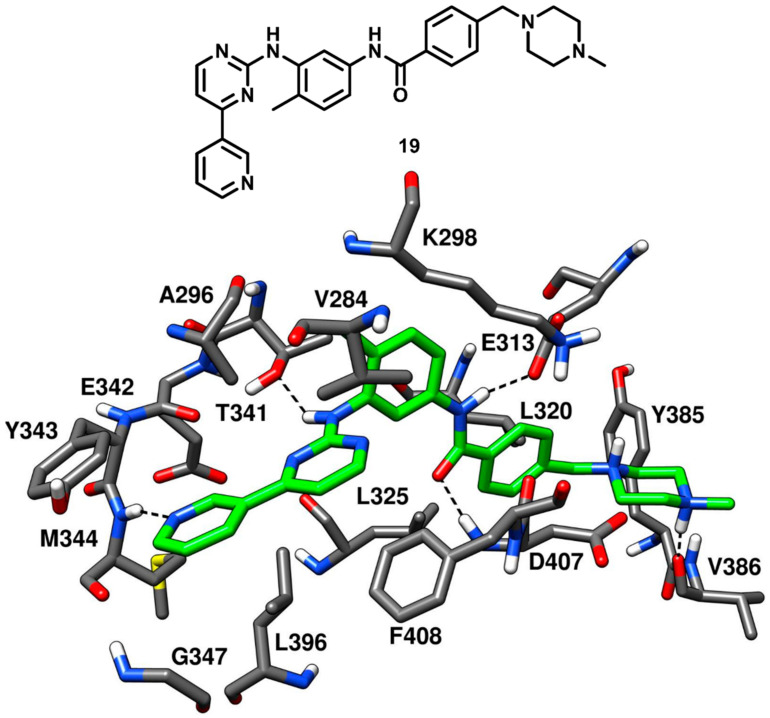
2D structure of **19** and X-ray structure of c-Src kinase in complex with **19** (PDB ID: 2OIQ). Active site residues of c-Src kinase and the enzyme–inhibitor H-bond interactions are shown.

As discussed in type-I inhibitors’ category, imatinib (compound **19**, Figure 17) is a potent inhibitor of Abl kinase but a weak inhibitor of c-Src kinase; however, the des-methyl analogue of imatinib (compound **2**, PDB ID 1Y57, Figure 4) had shown an impressive IC_50_ of 1.6 nM against c-Src kinase [43]. Seeliger and coworkers in 2007 co-crystallized compound **19** with c-Src kinase (Figure 17, PDB ID 2OIQ) and explored the structural basis of its affinity for c-Src, which could provide a useful rationale of its weak inhibitory activity [42]. Compound **19** was bound to the DFG-out inactive kinase conformation where an outward flip of F408 in the DFG pocket was observed, which categorized compound **19** as a DFG-out type-II inhibitor of c-Src. Compound **19** adopted an inverted binding orientation in the active site of c-Src with respect to the binding mode of the demethylated analogue **2**. The presence of a methyl group on the phenyl ring adjacent to the pyrimidine core of compound **19** determined steric repulsions with the hinge-region residues and the allocation of the methylated phenyl ring in the hydrophobic pocket I. The pyridine and pyrimidine rings of the inhibitor **19** occupied the adenine pocket of the ATP binding site. The nitrogen of the pyridine ring established one H-bond interaction with the backbone nitrogen of M344 whereas the pyrimidine ring was found to be in proximity with V284 of the N-terminal lobe and F408 of the DFG pocket, forming hydrophobic interactions with these residues. Moreover, the amino group of the pyrimidine moiety formed an H-bond with the gatekeeper residue T341. The benzamide group of the ligand occupied the DFG pocket and established key H-bonds with the E313 carboxylic group of the αC-helix and the backbone nitrogen of D407 from the DFG motif, while the adjacent phenyl ring showed van der Waals interactions mainly with the sidechains of L320 and L325. Finally, the piperazine tail of the inhibitor extended past the DFG pocket into the solvent exposed region, displayed an H-bond with the backbone oxygen of V386 and formed van der Waals contact primarily with Y385. The binding mode of compound **19** demonstrated that the presence of sterically bulky groups in the portion of the inhibitor occupying the adenine pocket prevented the formation of the classical H-bonding interactions in the hinge-region as compared to type-I inhibitors. This steric repulsion significantly changed the binding conformation of the inhibitor; however, the orientation of the benzamide moiety in the DFG pocket and the H-bonding interactions demonstrated by the amide linker with E313 and D407 were greatly exploited to develop novel DFG-out type-II ligands of c-Src.

**Figure 18:**
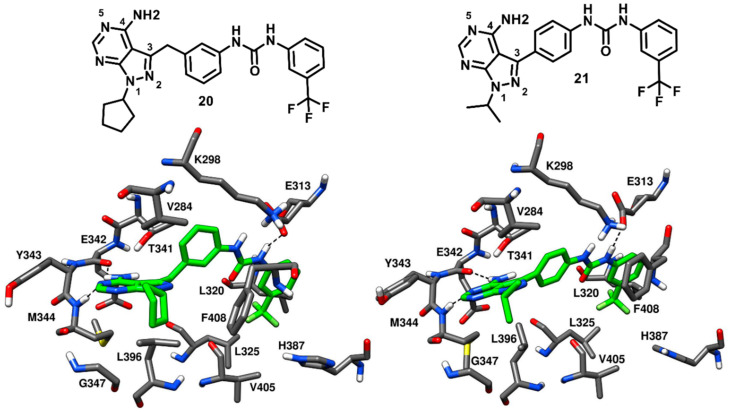
2D structures of **20** and **21**. X-ray structures of c-Src kinase in complex with **20** (PDB ID: 3EL7) and **21** (PDB ID: 3EL8). Active site residues of c-Src kinase and the enzyme–inhibitor H-bond interactions are shown.

Dar and coworkers in 2008 designed and synthesized a panel of pyrazolopyrimidine-based inhibitors, with reference to the binding mode of imatinib, that would bind to the inactive DFG-out conformation of c-Src kinase. The synthesized ligands showed IC_50_ values of c-Src inhibition from micromolar to low-nanomolar range. Two compounds of the series (**20** and **21**, Figure 18) were co-crystallized with c-Src (PDB ID: 3EL7 and 3EL8, respectively) and were both confirmed to act as type-II inhibitors, as they bound to the DFG-out inactive conformation of c-Src [73]. Unlike imatinib, both inhibitors showed a dual inhibitory effect on c-Src and Abl. Compound **20** showed to be less potent, with IC_50_ values of 0.480 µM and 2.087 µM against c-Src and Abl, respectively, whereas compound **21** demonstrated a higher activity, with IC_50_ values of 25 nM and 41 nM against c-Src and Abl, respectively. The pyrazolopyrimidine core of both compounds occupied the adenine pocket, forming one H-bond with the backbone nitrogen of M344 in the hinge-region, while the 4-amino group established an H-bond with the backbone oxygen of E342. The C-3 substituents of both ligands were twisted out of plane relative to the pyrazolopyrimidine ring, with the benzyl ring of **20** showing to better fit in the hydrophobic pocket I, which could contribute to its higher potency compared to **21**. The urea group of the ligands formed H-bonds with the carboxylic group of E313, whereas the trifluoromethyl phenyl ring of both inhibitors was well accommodated into the DFG pocket and displayed van der Waals contacts with the sidechains of L320, L325, H387 and V405, contributing to the binding affinity for the target. Hydrophobic interactions with V284 were also observed for the solvent exposed cyclopentyl and isopropyl groups of **20** and **21**, respectively. In addition, a lipophilic interaction between the cyclopentyl of **20** and F408 from the DFG motif, not observed for **21**, was noted and could further justify the higher activity of **20** compared to **21**. The distinctive feature of these pyrazolopyrimidine ligands is the rotation of their benzyl/phenyl ring away from the gatekeeper residue (similarly to the pyrimidine ring of imatinib). The extra space from the gatekeeper residue, created as a result of this rotation suggested that inhibitors **20** and **21** could bind to the clinically relevant T341M/T341I mutant c-Src and show promising results, which could further provide a platform for optimization studies against drug-resistant c-Src kinase.

**Figure 19:**
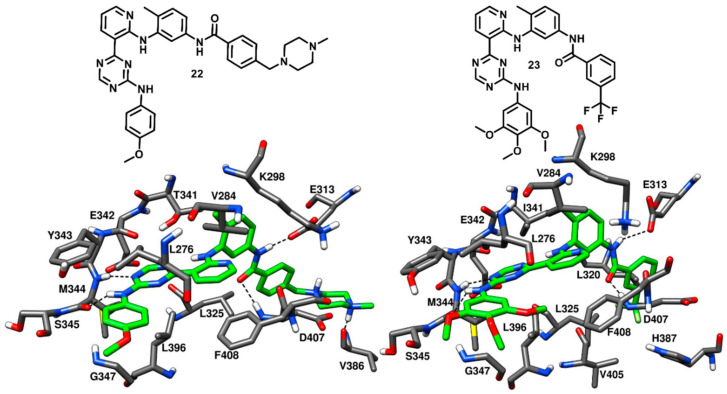
2D structures of **22** and **23**. X-ray structures of c-Src kinase in complex with **22** (PDB ID: 3G6G) and T341I c-Src in complex with **23** (PDB ID: 3G6H). Active site residues of c-Src kinase and the enzyme–inhibitor H-bond interactions are shown.

In 2009, Seeliger and coworkers identified a series pyridinyl-triazine inhibitors (also called as DSA compounds), that are based on the central scaffold of imatinib and were designed to bind c-Src with DFG-out kinase conformation. Unlike Imatinib, these compounds are nanomolar inhibitors of both c-Src and Abl with similar potencies. The most promising derivatives of the series, compounds **22** and **23** (Figure 19), were co-crystallized with wild type (PDB ID: 3G6G) and T341I mutant c-Src (PDB ID: 3G6H), respectively, and showed binding to the DFG-out kinase conformation as type-II inhibitors (Figure 19) [74]. Compounds **22** and **23** displayed IC_50_ values of 2.8 nM and 4.6 nM, respectively, against wild-type c-Src and IC_50_ values of 18 nM and 6.4 nM, respectively, against T341I mutant c-Src, thus demonstrating the retention of their inhibition potencies against the mutant enzyme, with an only two- to eightfold increase in IC_50_ values. The X-ray complexes of the ligands showed that the amino-group of their lateral aniline moieties formed an H-bond with the backbone oxygen of M344, while the triazine ring of both inhibitors formed another H-bond with the backbone nitrogen of M344. Moreover, the aniline moieties showed van der Waals contacts with hydrophobic pocket II residues, mainly with L276 and G347. Similarly to what observed for imatinib [42], the pyridine ring of both inhibitors established van der Waals interactions with the sidechains of V284 of N-terminal lobe and F408 of the DFG motif, the adjacent aromatic phenyl ring occupied hydrophobic pocket I interacting with the gatekeeper residue, while the amide linker was placed at the entrance of the DFG pocket and established key H-bonds with the carboxylic group of E313 of the αC-helix and the backbone nitrogen of D407 from the DFG motif. By comparing the X-ray structures of wild type and mutant c-Src in complex with **22** and **23**, respectively, it is possible to observe that the orientation of the central 4-methyl-*m*-diaminophenyl ring of **23**, which is placed in front of I341, is not perturbed by the larger side chain of the mutated gatekeeper residue, closely resembling the orientation observed for the same moiety of compound **22**. In fact, the binding mode of **23** was found to be comparable with that observed for **22** and this rationalizes the retention of inhibitory activity of **23** against T341I mutant c-Src with respect to wild type c-Src. Finally, the phenyl ring of **22** and the trifluoromethylphenyl ring of **23** occupied the DFG pocket. The phenyl ring of **22** established van der Waals contacts mainly with L325, whereas the trifluoromethylphenyl ring of **23** was surrounded by the side chains of L320, L325, H387, V405 and displayed strong van der Waals interactions. However, the terminal methylpiperazine ring of **22** extended past the DFG pocket into the solvent-exposed region and established an H-bond interaction with V386 of the C-terminal lobe.

Although the binding mode of **22** and **23** resemble that of imatinib, some dissimilarities can be observed in their structural orientation, which may contribute to their nanomolar binding affinity, in contrast with the weak affinity of imatinib against c-Src. The triazine and pyridine rings of **22** and **23** occupied the adenine pocket like the pyridine and pyrimidine groups of imatinib but showed different interactions. The pyridine in both **22** and **23** is *ortho*-substituted, while the equivalent pyrimidine in imatinib is *meta*-substituted. The different orientation of the pyridine and triazine ring in **22** and **23** led to a better accommodation of the two ligands into the adenine pocket, favored by stronger hydrophobic contacts with V284 and F408. Moreover, although **22** and **23** lacked the H-bond with the sidechain of the gatekeeper residue T341, the two ligands formed an H-bond with the hinge region, precisely with the backbone oxygen of M344, through the amino group of their terminal aniline rings. These considerations may justify the higher inhibitory activity of **22** and **23** with respect to imatinib.

**Figure 20:**
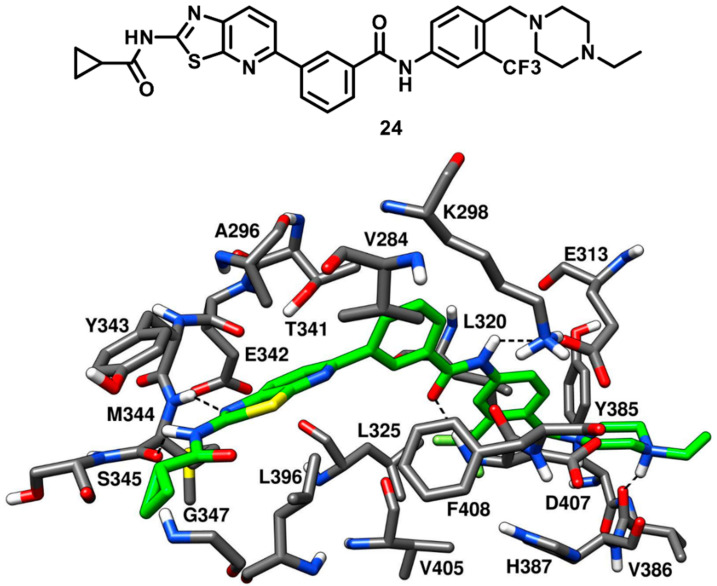
2D structure of **24**. X-ray structures of c-Src kinase in complex with **24** (PDB ID: 4AGW). Active site residues of c-Src kinase and the enzyme–inhibitor H-bond interactions are shown.

Weisberg and coworkers in 2010 developed a unique thiazolopyridine-based multitarget kinase inhibitor (**24**, Figure 20). The design of compound **24** involved a hybrid approach based on the type-I inhibitor dasatinib [38] and the type-II inhibitor nilotinib [75], which were both co-crystallized in complex with Abl kinase. The superimposition of the two ligand–protein co-crystal structures inspired the fusion of the hinge-interacting aminothiazole moiety of dasatinib and the N-(3-(trifluoromethylphenyl) benzamide moiety of nilotinib, which binds to the DFG-out conformation of the kinase. Compound **24** was thus designed as a type-II inhibitor, with the aim of developing a potent inhibitor of both wild-type and drug-resistant T341M/I mutant c-Src. Biochemical screening studies revealed that **24** was endowed with high inhibitory activity against a variety of wild-type and mutant forms of kinases. To confirm the proposed binding mode resulting from the hybrid design, compound **24** was co-crystallized with c-Src (PDB ID 4AGW, Figure 20) [76]. As expected, this inhibitor was bound to the inactive DFG-out kinase conformation and, interestingly, displayed five H-bonding interactions with the binding site residues of c-Src. The thiazolopyridine ring of compound **24** occupied the adenine pocket, the thiazole nitrogen formed one H-bond with the backbone nitrogen of M344 while the fused pyridine ring showed hydrophobic interactions with A296 and L396. The amino group of the cyclopropylamide of the inhibitor formed another H-bond with the backbone oxygen of M344, while the cyclopropyl moiety was solvent-exposed. The DFG-out conformation of c-Src kinase allowed the trifluoromethylphenyl ring of the inhibitor to occupy the DFG pocket, whereas the amide linker connecting phenyl and trifluoromethyl phenyl ring formed important H-bonds with the carboxylic group of E313 of the αC-helix and the backbone nitrogen of D407 from the DFG motif, which represented the prototypical feature of imatinib-based compounds. The phenyl of the benzamide moiety of the inhibitor displayed an edge-to-face π-π stacking with F408 of the DFG motif, while the trifluoromethylphenyl substituent demonstrated strong van der Waals contacts with L320, L325, H387 and V405. The ethylpiperazine tail of the inhibitor was allocated past the DFG pocket into the solvent-exposed area, displaying hydrophobic contacts with Y385 and forming an H-bond with the backbone oxygen of V386. Similarly to inhibitors **22** and **23**, the loss of an H-bond with the sidechain of T341 observed for imatinib is compensated by an additional H-bond formation between the amino group of the cyclopropylamide and the backbone oxygen of M344. Molecular modeling studies performed with compound **24** suggested the possibility that mutant c-Src with larger gatekeeper residues (T341I/M) could accommodate the ligand without affecting its binding mode. In fact, **24** was reported to inhibit the proliferation of Ba/F3 cells transfected with wild type c-Src and both T341I and T341M mutant c-Src. The EC_50_ values for compound **24** were determined to be 0.19 µM, 0.29 µM, and 0.15 µM for c-Src, T341I c-Src and T341M c-Src respectively. On the contrary, the nanomolar c-Src inhibitor dasatinib (**1**) showed an EC_50_ value < 10 nM for wild type c-Src but completely lost activity for both T341I and T341M mutant c-Src (EC_50_ > 10 µM). This experimental data confirmed that the thiazolopyridine compound **24** is a potent inhibitor of both wild-type and drug-resistant c-Src kinase (T341I/M).

**Figure 21:**
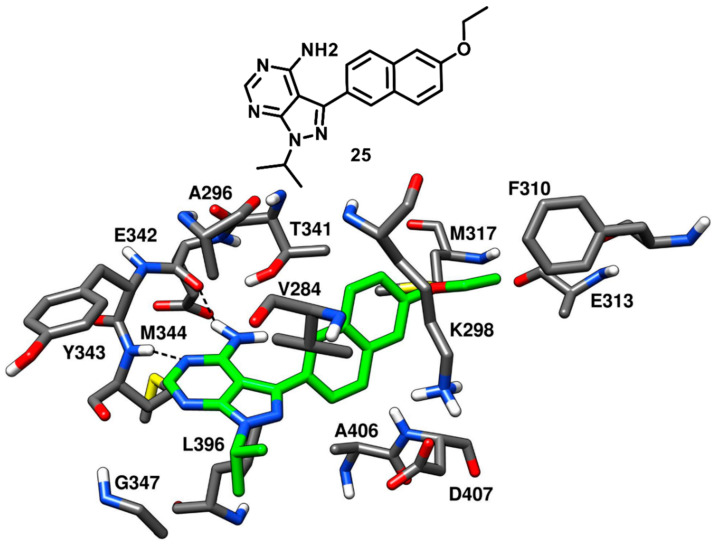
2D structure of **25**. X-ray structures of c-Src kinase in complex with **25** (PDB ID: 3UQF). Active site residues of c-Src kinase and the enzyme–inhibitor H-bond interactions are shown.

Larson and coworkers in 2012 developed a series of pyrazolopyrimidine-based apicomplexan CDPK1 inhibitors and reported a comparison analysis of the ligand–protein interactions observed in X-ray structures of CDPK1 and c-Src in complex with inhibitors. SAR studies were then performed with the aim to explore the pyrazolopyrimidine scaffold and a series of inhibitors comprising either isopropyl or 4-piperidinemethyl groups at 1-positon [77] and various substituents at the 3-position of the pyrazolopyrimidine core was synthesized. One of the best compounds of the series (**25**, Figure 21) was co-crystallized with c-Src kinase (PDB ID: 3QUF) and demonstrated an IC_50_ of 190 nM [78]. Compound **25** was bound to inactive c-Src with CHO conformation and could be therefore categorized as a CHO-type-II inhibitor of c-Src. The 4-aminopyrazolopyrimidine core laid on the adenine pocket of ATP binding site, forming hydrophobic interactions with A296 and L396, and displayed classical H-bond interactions with the hinge-region residues: one H-bond with the backbone nitrogen of M344 and one with the backbone oxygen of E342. The highly hydrophobic and bulky ethoxynaphthalene substituent of the pyrazolopyrimidine ring well occupied hydrophobic pocket I, forming hydrophobic interactions with the sidechains of K298 and T341 residues and led to the disruption of the key salt bridge interaction between K298 of the N-terminal lobe and E313 of the αC-helix. Furthermore, the terminal ethoxy group was encircled by the sidechains of F310, E313 and M317, forming van der Waals contacts with these residues. Finally, the isopropyl group of the ligand was solvent-exposed but showed van der Waals contacts with V284. SAR studies highlighted that the naphthyl substituent, which was projected deeply towards the αC-helix, largely contributed to the activity of the ligand against c-Src. In fact, a synthesized analogue lacking the naphthyl substituent (**26**, Figure S4) showed a drastic loss of inhibition potency for c-Src, with an IC_50_ > 10 µM. The X-ray structure of c-Src in complex with compound **26** (PDB ID: 3UQG) demonstrated that the absence of the large C-3 substituent prevented the ligand to interfere with the interaction between K298 and E313, and the compound bound to the αC-helix-in active conformation of c-Src as a type-I inhibitor. The pyrazolopyrimidine moiety of **26** displayed H-bonding interactions in the hinge-region similar to **25**, while the solvent-exposed N-1 isopropyl substituent of **25** was replaced with 4-piperidinemethyl substituent, which did not make any contact with active site residues.

**Figure 22:**
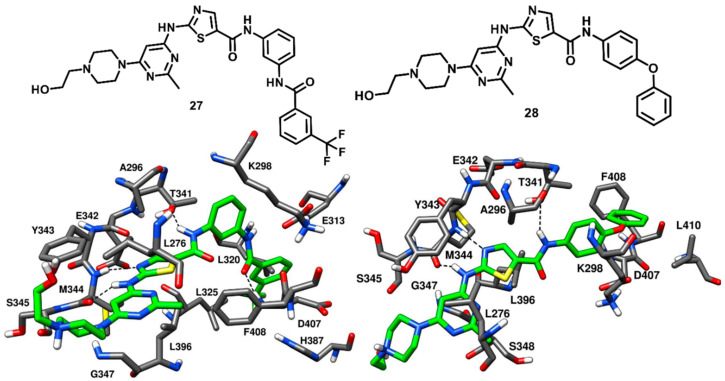
2D structures of **27** and **28**. X-ray structures of c-Src kinase in complex with **27** (PDB ID: 4YBJ) and **28** (PDB ID: 4YBK). Active site residues of c-Src kinase and the enzyme–inhibitor H-bond interactions are shown.

Kwarcinski and coworkers in 2015 adopted a hybrid design strategy to develop dasatinib analogues, that could bind to either CHO or DFG-out inactive kinase conformation. An overlay of the X-ray structures of c-Src bound to datasinib (compound **1**, PDB ID: 3G5D, Figure 3) and imatinib (compound **19**, PDB ID: 2OIQ, Figure 17) suggested that the addition of a benzamide group to the terminal phenyl ring of dasatinib could result in a potent type-II derivative. Two analogues of dasatinib were synthesized, and the most active compound **27**, which demonstrated an impressive binding affinity, with a K_d_ value of 0.52 nM, was co-crystallized with c-Src (PDB ID: 4YBJ, Figure 22) [69]. Compound **27** demonstrated binding to the inactive kinase conformation, with the portion belonging from dasatinib accommodated into the ATP binding site and that derived from imatinib located into the DFG pocket of the activation loop. An outward flip of F408 of the DFG motif was observed in the inactive kinase conformation, which classified compound **27** as a DFG-out type-II inhibitor. Compound **27** showed the two classical H-bond interactions in the hinge-region of the ATP-binding site; the amino group of the 2-aminothiazole moiety formed an H-bond with the backbone oxygen of M344, while the nitrogen of the thiazole ring formed another H-bond with the backbone nitrogen of M344. The amide nitrogen of the thiazole-5-carboxamide established a crucial H-bond with T341, which could provide selectivity for c-Src. The 2-methyl pyrimidine ring of **27** showed van der Waals contacts with L276, G347 and L396, while the thiazole ring of the inhibitor displayed van der Waals interactions with A296 and L396. The carbonyl oxygen of the benzamide moiety of the inhibitor displayed an H-bond with the backbone nitrogen of D407 in the DFG pocket, whereas the trifluoromethyl ring of the inhibitor occupied the DFG pocket and formed van der Waals contacts with L320, L325, H387 and V405.

A similar design strategy was applied for the development of few more dasatinib analogues as type-II CHO inhibitors of c-Src. An overlay of dasatinib-c-Src complex with a group of known CHO inhibitors of other kinases (such as EGFR, HER2, MET and BTK) [79,80] suggested that addition of a *para*-phenoxy group to dasatinib could lead to a potent type-II CHO analogue. Two dasatinib-CHO analogues were synthesized and the most active compound (**28**) showed high binding affinity for the kinase, with a K_d_ value of 15 nM, and was co-crystallized with c-Src (PDB ID: 4YBK, Figure 22). Compound **28** was bound to the inactive kinase conformation, with type-II CHO mode of inhibition. Compound **28** displayed H-bonding interactions with the backbone of M344 in the hinge-region and with the gatekeeper residue in the hydrophobic pocket I, similarly to compound **27**. The inhibitor penetrated very deeply into hydrophobic pocket I and the αC-helix was projected outwards, undergoing a big rotation of around 5 Å from its original position, with the consequent disruption of the key salt bridge interaction between K298 and E313. The phenoxy group of compound **28** was not able to access the DFG pocket of the activation loop, but occupied an empty space emerged from the outward movement of the αC-helix and formed a T-shaped π-π stacking with the phenyl ring of F408. More van der Waals contacts were noted for the phenoxy group of the inhibitor with the sidechains of K298 and L410. Both compounds **27** and **28**, together with dasatinib, were subjected to competition binding assays and tested on a panel of 124 non-mutant kinase constructs at the concentration of 500 nM, in order to assess the selectivity of the two ligands. While compound **27** showed a selectivity profile comparable to dasatinib, compound **28** showed to be much more selective, demonstrating affinity for a reduced number of kinases (with an S_35_ score of 0.05 compared to the S_35_ score of 0.21 obtained for dasatinib).

### 2.5. Type-III and Type-III-Derived Inhibitors

**Figure 23:**
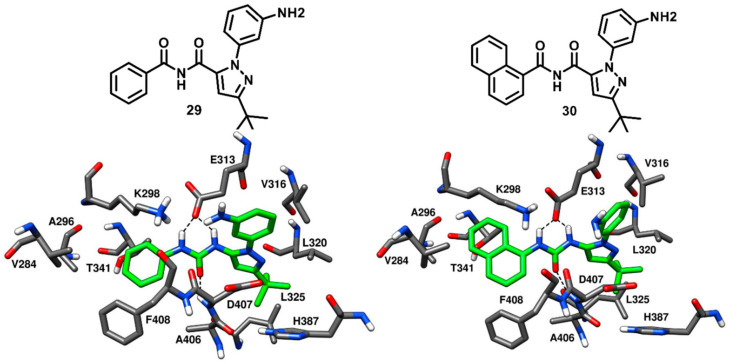
2D structures of **29** and **30**. X-ray structures of c-Src kinase in complex with **29** (PDB ID: 3F3U) and **30** (PDB ID: 3F3T). Active site residues of c-Src kinase and the enzyme–inhibitor H-bond interactions are shown.

Emergence of drug resistance is a fundamental challenge in the development of kinase inhibitors for prolonged treatments. As discussed before, the most common mutation occurs at the gatekeeper position in the hinge region, where the small amino acid T341 is exchanged with a larger hydrophobic residue (I or M) [35,36]. Some of the type-I and type-II inhibitors demonstrated drug resistance due to the steric clashes with the mutated gatekeeper residues. In this context, another important class of c-Src ligands is represented by type-III inhibitors, which exclusively bind to the less-conserved allosteric site in the DFG pocket of the inactive c-Src conformation and do not interact with hinge-region and ATP binding site. For this reason, type-III inhibitors form either no or limited interactions with the gatekeeper residue. Though allosteric type-III inhibitors often show mid-micromolar inhibitory potency, they have been found to overcome drug resistance caused by mutations at the gatekeeper position that induce a steric clashes and prevents inhibitors from effectively binding to the hinge region. Moreover, the binding mode of type-III inhibitors have been employed for the development of potent type-I and type-III hybrid inhibitors that are capable of circumventing drug resistance. Several pyrazoloureas were known to be potent type-III binders of p38α kinase with affinities in the low nanomolar range [81,82]. Inspired by these compounds, Simard and coworkers in 2009 identified four pyrazolourea derivatives as the first type-III inhibitors of c-Src by screening a compound library. Enzyme activity assays confirmed that two out of these four hit compounds (**29** and **30**, Figure 23) inhibited c-Src kinase activity with IC_50_ values in the mid-micromolar range (Table S4). Compounds **29** and **30** demonstrated an IC_50_ of 32.1 µM and 64.1 µM against c-Src, respectively, and were co-crystallized with c-Src kinase (PDB IDs: 3F3U and 3F3T, respectively, Figure 23) to confirm the type-III allosteric binding mode. To our knowledge, these are the first and only reported crystal structures of c-Src kinase in complex with type-III inhibitors. Both ligands were bound to the inactive kinase conformation and were located outside the ATP binding pocket, which is a prototypical character of type-III kinase inhibitors. An outward flip of F408 was observed in the DFG pocket, thus characterizing the two ligands as DFG-out type-III inhibitors of c-Src. Both the phenyl and naphthyl ring of **29** and **30**, respectively, occupied hydrophobic pocket I, with the naphthyl ring of **30** being closer to the gatekeeper residue. Furthermore, both substituents were found to be in the proximity of F408 of the DFG motif, forming hydrophobic interactions with this residue. The urea moiety of the ligands formed H-bonds with the carboxylic group of E313 of the αC-helix and the backbone nitrogen of D407 in the DFG pocket. The C-3 tert-butyl substituted pyrazole ring of both inhibitors was accommodated into the DFG pocket, with the tert-butyl group displaying van der Waals contacts with L320, L325 and H387. The terminal 3-aminophenyl ring exhibited van der Waals interactions predominantly with E313 and V316 [37]. 

Despite the micromolar potency of compound **29** and **30**, the identification of type-III c-Src inhibitors encouraged the development of potent analogues active against drug-resistant c-Src (T341M/I). Similar to the hybrid design approach employing type-I and type-II inhibitors discussed before, type-I/II and type-III hybrid design demonstrated to offer a profitable opportunity for scaffold development to circumvent drug resistance. In this context, Getlik and coworkers in 2009 employed a fragment-based design strategy to develop hybrid molecules from type-III pyrazolourea inhibitors and type-I quinazoline inhibitors. Activity assays using drug resistant c-Src variant (T341M) [83] performed for compound **29** and **30** showed that the presence of the bulkier gatekeeper residue had no effect on the potency of compound **29**, while compound **30** dramatically lost its activity. The X-ray structures suggested that compound **30** was likely to be impeded by steric repulsions with the larger gatekeeper residue due to the presence of its bulky naphthalene moiety, which was placed in close proximity to T341, while the phenyl moiety of compound **29**, which was more distant from the gatekeeper residue, was unaffected by its mutation. The pyrazolourea ligand **29** was thus used as a reference scaffold for hybrid-inhibitor development. 

The covalent 4-(phenylamino)quinazoline inhibitor (compound **31a**, Figure S5), previously developed by the same research group was used as the second scaffold. The quinazoline inhibitor showed IC_50_ values of 0.23 µM and 5.71 µM for wild-type and drug-resistant (T341M) c-Src, respectively [36]. The X-ray structure of c-Src in complex with compound **29** (PDB ID: 3F3U) was superimposed with the c-Src-**31a** co-crystal structure (PDB ID: 2QLQ), which showed that the aniline moieties of both inhibitors were well aligned to each other in the vicinity of the gatekeeper residue. The alignment suggested that more potent inhibitors could be generated by fusing the scaffolds of the two compounds via 1,3 or 1,4-disubstituted aryl linkage. Docking studies suggested that 1,4-para hybrid compounds would be better accommodated into the c-Src active site, provided by optimal geometry to avoid steric clashes with the larger gatekeeper residue. Thus, the 1,4-substitution pattern was principally exploited to design and synthesize a small library of compounds with a proposed type-II inhibitor binding mode. However, few 1,3-disubstituted hybrids were also designed and synthesized for comparison studies. The synthesized 1,4-substituted hybrids showed at least a fourfold improvement in activity compared to the inhibition potencies of both parent ligands. Most importantly, 1,4-substituted hybrids showed no loss of potency against the drug-resistant T341M c-Src while 1,3-substituted hybrids demonstrated weak inhibition of wild-type c-Src kinase and complete loss of activity against drug-resistant T341M c-Src. 

**Figure 24:**
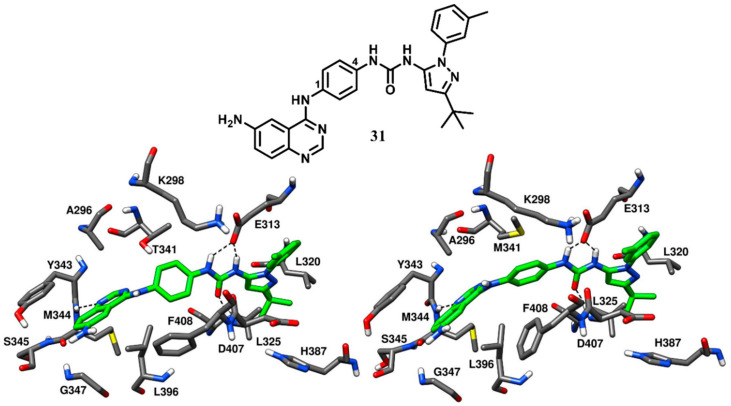
X-ray structure of wild type c-Src (PDB ID: 3F3V) and drug-resistant c-Src (PDB ID: 3F3W) in complex with **31**. Active site residues of c-Src kinase and the enzyme–inhibitor H-bond interactions are shown.

One of the 1,4-substitued hybrid inhibitors (compound **31**, Figure 24) was co-crystallized with both wild-type and drug-resistant T341M c-Src (PDB ID: 3F3V and 3F3W, respectively) to get deeper insights into its binding mode. The IC_50_ values of compound **31** were found to be 21 nM and 34 nM against wild-type c-Src and drug-resistant T341M c-Src, respectively [71]. Compound **31** was bound to the DFG-out inactive conformation of the kinase, where an outward flip of F408 of the DFG motif was observed. The quinazoline core of compound **31** occupied the ATP-binding site of both wild-type and drug-resistant c-Src, showing classical H-bond interactions with the backbone nitrogen of M344 in the hinge-region. The central phenyl ring that links the quinazoline and pyrazolourea scaffolds of the parent compounds was sandwiched between the gatekeeper residue and the side chain of F408 of the DFG motif, forming π-π staking interactions with this latter residue. The presence of the larger gatekeeper residue in the T341M c-Src forced the central phenyl ring of the inhibitor to rotate by 90 degrees in order avoid steric clashes with the side chain of M341. The side of chain of F408 was also rotated by 90 degrees to conserve the edge-to-face π-π stacking with the phenyl ring of the inhibitor [84]. The urea group of the inhibitors displayed H-bond interactions with the carboxylic group of E313 and the backbone nitrogen of D407, while the adjacent pyrazole ring was placed within the DFG pocket in both wild-type and T34IM mutant c-Src, as observed for the parent compounds **29** and **30**. The C-3 tert-butyl group of the inhibitor showed van der Waals interactions with L320, L325 and H387, while the 3-amino phenyl ring displayed van der Waals contacts with the sidechains of E313 and V316.

Docking results suggested that the 1,3-substituted hybrid (compound **32**, Figure S5) was tolerated in the wild-type c-Src. However, in the drug-resistant T341M c-Src, the rotation of the central phenyl core in compound **32** was not possible without disrupting the binding orientation of either the quinazoline or the pyrazolourea moiety. The decreased inhibitor flexibility could result in the loss of H-bonds or the displacement of the pyrazolourea from the allosteric site, in order to adapt the ligand conformation to the larger gatekeeper residue. This explains why compound **32** was not able to bind the mutated c-Src. Therefore, the 1,4-substitution pattern employed in the development of quinazoline-pyrazolourea hybrid inhibitors proved to be a robust rational to overcome T341M c-Src drug resistance with significantly improved potency.

## 3. Conclusions

During the last two decades, great efforts have been made for the design and development of c-Src and SFKs inhibitors as potential antitumor agents. Undoubtedly, the well-documented role of c-Src in cancer development and progression made this kinase a very appealing target for anticancer therapy. The unceasing and continuous interest for c-Src in the medicinal chemistry field is demonstrated by the plethora of compounds endowed with c-Src inhibitory activity that have been identified and reported in literature, few of which have been accepted as actual drugs or as clinical trial candidates. A considerable number of these ligands have been co-crystallized with c-Src, revealing important structural information for the understanding of the key elements necessary for achieving high c-Src inhibitory potency and those that may be exploited in order to address the issues of selectivity and drug resistance. By inspecting the different X-ray structures of c-Src kinase in complex with small-molecule inhibitors herein illustrated, and analyzing the main ligand–protein interactions observed therein, it emerges that the main features characterizing a potent c-Src inhibitor are represented by the proper occupancy of the different regions of the kinase binding site and the presence of further H-bonds in addition to the key interactions with the hinge region residues. These represent two different aspects that can be balanced for obtaining a highly potent c-Src inhibition. In fact, although the presence of all these features may allow to achieve very high inhibitory activities, as for compounds **1** (dasatinib) and **6** that showed subnanomolar potency, compound **2** (IC_50_ = 1.6 nM) and **15** (saracatinib, IC_50_ = 2.7 nM) show that a good shape complementarity with the kinase binding site can make up for the lack of additional H-bonds. On the other hand, compounds with lower molecular weight such as **10** and **16** (with IC_50_ values of 14 and 15 nM, respectively) demonstrate that the occupancy of hydrophobic pocket I and the presence of an H-bond with E313 and T341, respectively, in addition to the interactions with the hinge region, can be enough to allow a considerably high inhibitory potency. Moreover, water-mediated H-bonds with residues located next to hydrophobic pocket I, as observed for compound **9** (bosutinib, IC_50_ = 1.2 nM) showed to represent an important aspect to take into account not only for inhibitory potency but also for selectivity. If the above reported considerations seem to be valid for type-I and CHO type-II inhibitors, they seem to be less valid for DFG-out type-II ligands, which often form many different H-bonds and show interactions with additional regions of the kinase binding site (such as the DFG-pocket) but maintain inhibitory potencies comparable with ligands showing fewer H-bonds and/or hydrophobic interactions. This can be observed comparing the DFG-out ligands **22** and **23** (with IC_50_ values of 2.8 and 4.6 nM, respectively) with the type-I inhibitor **2**, which are all derivatives of imatinib, and comparing the DFG-out inhibitors **20** and **21** (with IC_50_ values of 480 and 25 nM, respectively) with the type-I ligand **10**, which can be considered as their parent compound. However, DFG-out type-II inhibitors showed to represent a powerful tool for overcoming drug resistance. In fact, DFG-out ligands often present aromatic moieties able to properly fill hydrophobic pocket I without closely interacting with the gatekeeper residue, so that its mutation into bulkier residues does not significantly perturb the binding mode of these inhibitors. Valuable examples of these type of ligands overcoming drug resistance are represented by compounds **22** and **23**, which showed similar inhibitory activities against wild type and T341I mutant c-Src, as well as **24** and **31**, which displayed comparable inhibitory potency against wild type and T341M mutant c-Src. In this context, exploiting the structural knowledge about type-III inhibitors, such as compounds **29** and **30**, which only allosterically bind to c-Src, for the design of new hybrid ligands (like **31**) seems to represent a fruitful strategy to fight drug resistance. Finally, the data herein reported suggest that in order to account for kinase selectivity, drug design efforts should be focused on the development of CHO type-II inhibitors and/or on the exploration of the region of space adjacent to hydrophobic pocket I of c-Src binding site. In fact, the CHO ligand saracatinib (compound **15**) proved to be remarkably selective over a variety of non-SFK kinases and, out of the two type-II derivative of dasatinib **27** and **28**, the CHO inhibitor **28** showed a considerably higher selectivity with respect to dasatinib, while the DFG-out ligand **27** displayed a selectivity profile similar to the parent compound. Moreover, the type-I inhibitor **9**, which formed a network of water-bridged H-bonds with residues adjacent to hydrophobic pocket I, often involved in interactions with type-II CHO ligands, proved to be selective over non-SFK kinases, suggesting that particular attention should be paid to that region of c-Src binding site for addressing kinase selectivity. Overall, we believe that the information derived from the analysis of the X-ray structures herein presented provide the reader with useful guidelines for the design of new potent c-Src inhibitors and for addressing drug-resistance and selectivity issues.

## Supplementary Materials

The following are available online at https://www.mdpi.com/2072-6694/12/8/2327/s1, Figure S1: X-ray structure of mutant c-Src in complex with compound 9, Figure S2: Chemical structure of compound 11a, Figure S3: X-ray structure of c-Src in complex with compounds 17 and 18, Figure S4: X-ray structures of c-Src kinase in complex with compound 26, Figure S5: Chemical structures of compounds 31a and 32, Table S1: Summary of the different types of c-Src inhibitors discussed, Table S2: Index of type-I inhibitors co-crystallized with c-Src kinase discussed, Table S3: Index of type-II inhibitors co-crystallized with c-Src kinase discussed, Table S4: Index of type-III inhibitors co-crystallized with c-Src kinase discussed.
