# Supplementary material for: Three-Dimensional Interactions Analysis of the Anticancer Target c-Src Kinase with Its Inhibitors
Source: Cancers (Basel). 2020 Aug 18;12(8):2327. doi: 10.3390/cancers12082327 (PMC7466017; doi:10.3390/cancers12082327)
Supplement: Supplementary file 1 [file cancers-12-02327-s001.pdf]

Supplementary Materials

Three-Dimensional Interactions Analysis of the Anticancer Target c-Src Kinase with Its Inhibitors

Vibhu Jha, Marco Macchia, Tiziano Tuccinardi and Giulio Poli

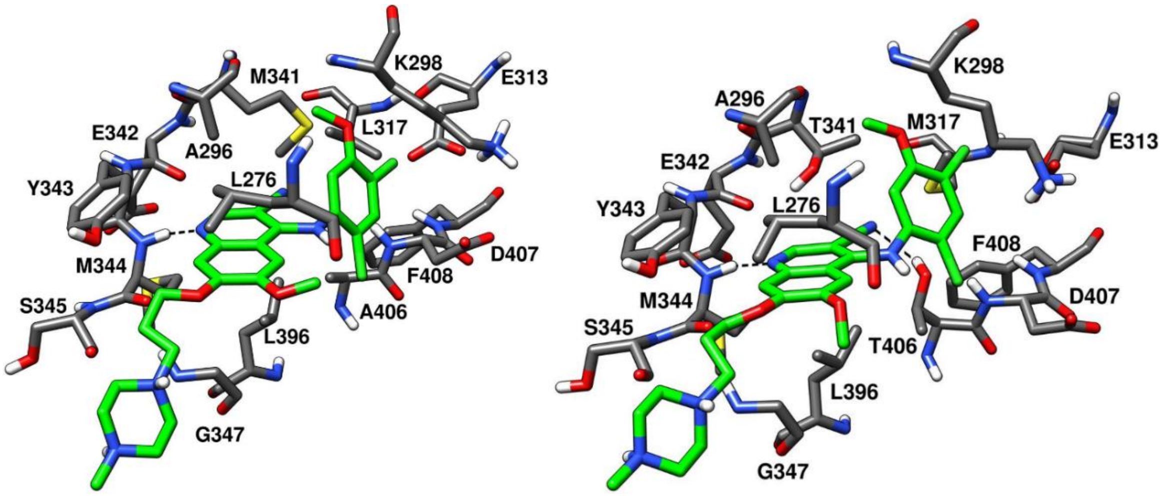

Figure S1. X-ray structure of T341M-M317L c-Src (PDB ID: 4MXZ) and A406T c-Src (PDB ID: 4MXX) in complex with **9**. Active site residues of c-Src kinase and the enzyme-inhibitor H-bond interactions are shown.

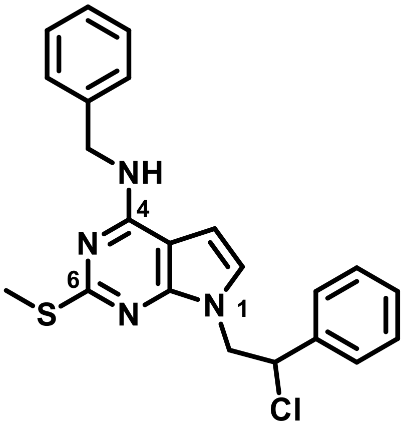

11a

Figure S2. Chemical structure of compound **11a**.

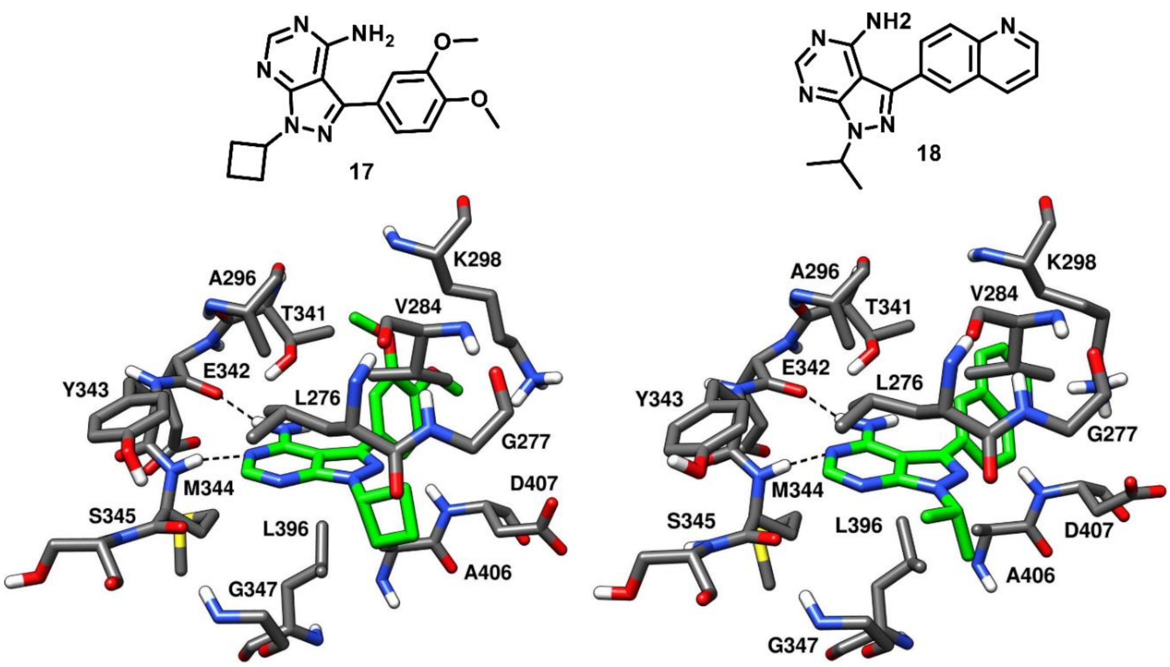

Figure S3. Structures of **17** and **18**. X-ray structures of c-Src kinase in complex with **17** (PDB ID: 3EN5) and **18** (PDB ID: 3EN6). Active site residues of c-Src kinase and the enzyme-inhibitor H-bond interactions are shown.

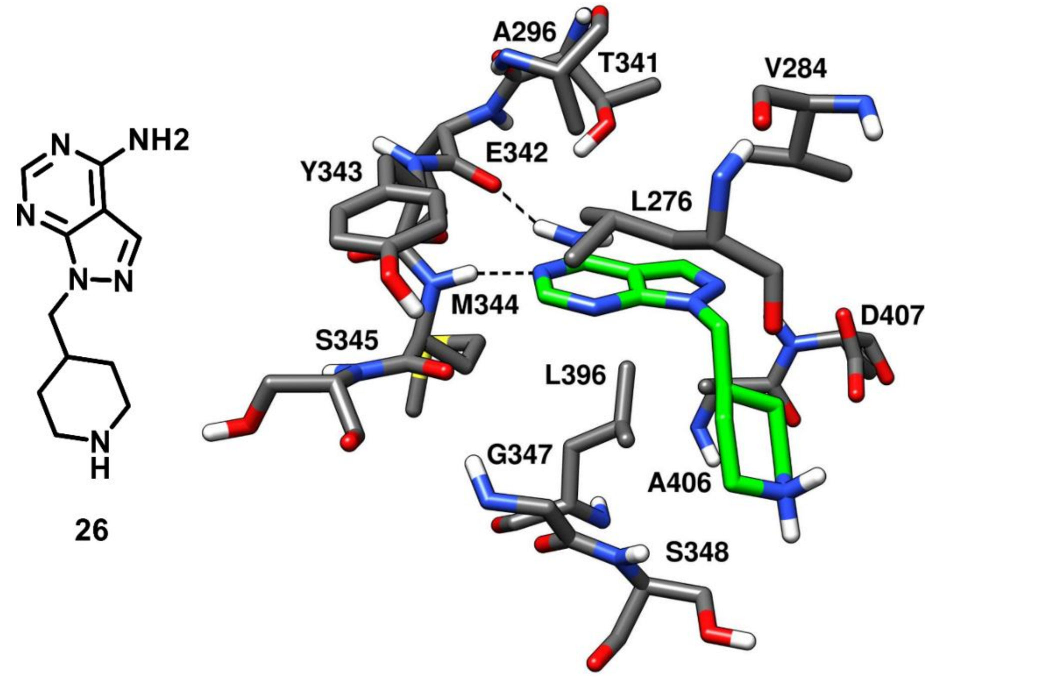

Figure S4. Structure of **26**. X-ray structures of c-Src kinase in complex with **26** (PDB ID: 3UQG). Active site residues of c-Src kinase and the enzyme-inhibitor H-bond interactions are shown.

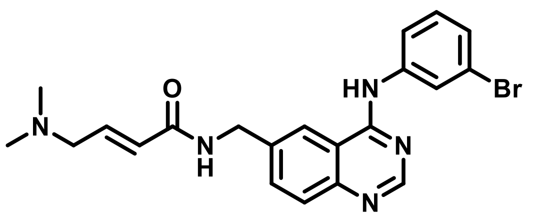

31a

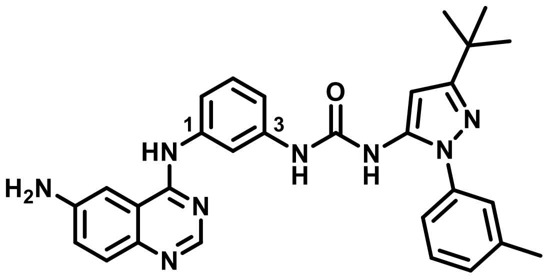

32

Figure S5. 2D-structure of type-I quinazoline inhibitor (**31a**) and 1,3 substituted inhibitor (**32**).

Table S1. Summary of the different types of c-Src inhibitors discussed; the structural features of the kinase conformation associated with each inhibitor type are reported.

Inhibitor Type	State of Kinase	Kinase Conformation Features	Number of Co-Crystal Structures Described
Type-I	Active	DFG-in, αC-helix-in	17 (compounds **1-14, 26**)
DFG-out Type-II	Inactive	DFG-out, αC-helix-in	8 (compounds **19-24, 27, 31**)
αC-helix-out Type-II	Inactive	DFG-in, αC-helix-out	6 (compounds **15-18, 25, 28**)
Type-III inhibitors	Inactive	DFG-out, αC-helix-in	2 (compounds **29, 30**)

Table S2. Index of type-I inhibitors of c-Src kinase co-crystallized with the protein discussed.

Inhibitor	PDB ID	Figure	2D-Structure	Biological Activity[a]	Binding Pocket Occupancy	Ref.
1 (Dasatinib, BMS-354825)	3G5D	3	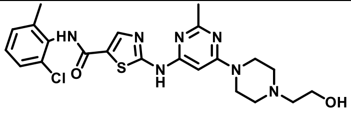	0.50 nM (IC_{50})	adenine pocket, hydrophobic pocket I and II	[38]
2	1Y57	4	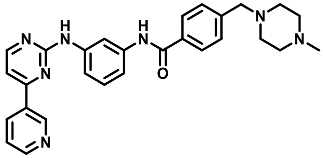	1.6 nM (IC_{50})	adenine pocket, hydrophobic pocket II, ribose pocket	[43]
3 (CGP77675)	1YOL	5	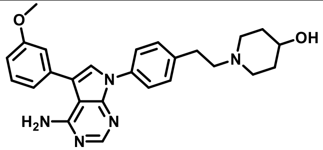	5-20 nM (IC_{50})	adenine pocket, hydrophobic pocket I, ribose pocket	[20]
4 (Ruxolitinib)	4U5J	6	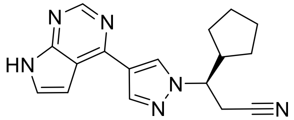	2.8 µM (IC_{50})	adenine pocket, hydrophobic pocket II	[47]

5 (Purvanalol A)	1YOM	7	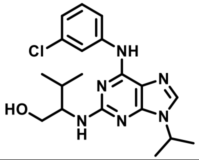	0.24 μM (IC_{50})	adenine pocket, hydrophobic pocket II, ribose pocket	[20]
6 (AP23464)	2BDJ	8	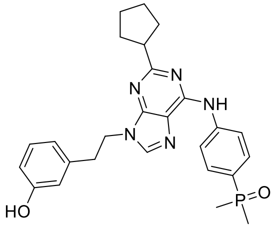	0.45 nM (IC_{50})	adenine pocket, hydrophobic pocket I and II, ribose pocket,	[50]
7 (AP23451)	2BDF	8	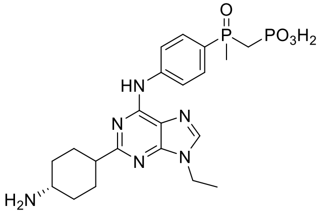	67 nM (IC_{50})	adenine pocket, hydrophobic pocket II, ribose pocket	[50]
8	4FIC	9	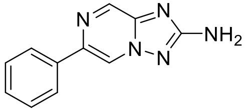	20 μM (IC_{50})	adenine pocket, hydrophobic pocket II	[52]
9 (Bosutinib)	4MXO	10	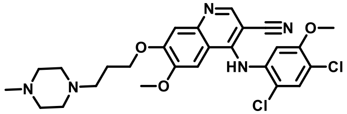	1.2 nM (IC_{50}) 0.73 nM (K_d)	adenine pocket, hydrophobic I and pocket II	[55]
	4MXZ (T341M-M317L)	S1		22.2 nM (K_d) for T341M/M317L Src		
	4MXX (A406T)	S1		29 nM (K_d) for A406T Src		
10	3EN4	11	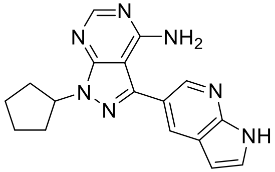	14 nM (IC_{50})	adenine pocket, hydrophobic pocket I, ribose pocket	[66]

11	4O2P	12	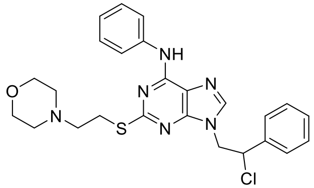	0.21 μM (K_i)	adenine pocket, hydrophobic pocket I and II, ribose pocket,	[64]
12	3F6X	13	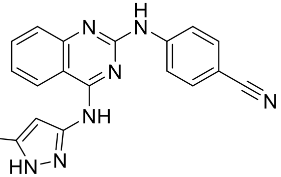	0.500 μM (K_d)	adenine pocket, hydrophobic pocket II, ribose pocket,	[65]
13	5D12	14	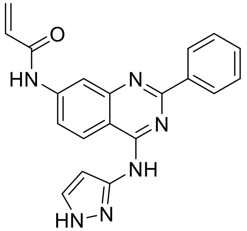	0.211 μM (IC_{50})<br>65 nM (IC_{50}) for T341M Src<br>43 nM (IC_{50}) for T341M/S348C Src	adenine pocket, hydrophobic pocket II, ribose pocket,	[67]
14	5D10	14	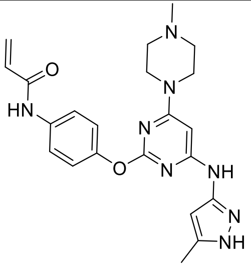	0.218 μM (IC_{50})<br>0.107 μM (IC_{50}) for T341M Src<br>72 nM (IC_{50}) for T341M/S348C Src	adenine pocket, hydrophobic pocket II, ribose pocket	[67]
26	3UQG	S4	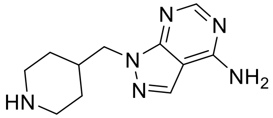	>10 μM (IC_{50})	adenine pocket	[78]

[a]Binding affinities are expressed as IC_{50}, K_d or K_i values. The biological activity data for mutant c-Src are specified individually (where applicable), while the unspecified binding affinities correspond to wild-type c-Src.

Table S3. Index of type-II inhibitors of c-Src kinase co-crystallized with the protein discussed.

Inhibitor	PDB ID	Figure	2D-Structure	Biological Activity[a]	Binding Pocket Occupancy (with Mode of Inhibition)	Ref.
15 (Saracatinib, AZD0530)	2H8H	15	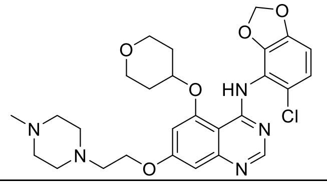	2.7 nM (IC_{50})	adenine pocket, hydrophobic pocket I and II, ribose pocket (αC-helix-out)	[71]
16	3EN7	16	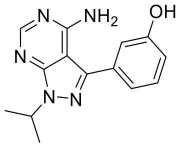	15 nM (IC_{50})	adenine pocket, hydrophobic pocket I, ribose pocket (αC-helix-out)	[66]
17	3EN5	S3	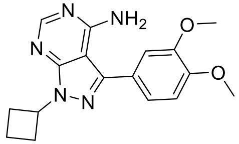	0.360 μM (IC_{50})	adenine pocket, hydrophobic pocket I, ribose pocket (αC-helix-out)	[66]
18	3EN6	S3	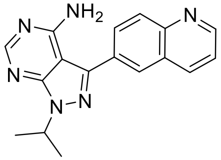	0.235 μM (IC_{50})	adenine pocket, hydrophobic pocket I, ribose pocket (αC-helix-out)	[66]
19 (Imatinib)	2OIQ	17	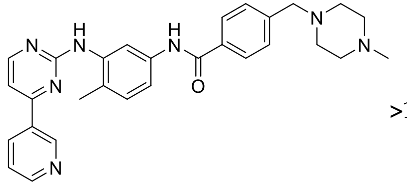	>100 μM (IC_{50})	adenine pocket, DFG pocket, hydrophobic pocket I (DFG-out)	[42]
20	3EL7	18	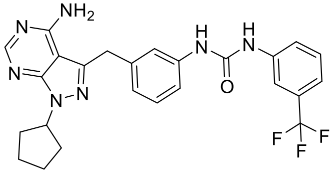	0.480 μM (IC_{50})	adenine pocket, DFG pocket, hydrophobic pocket I and II (DFG-out)	[73]

21	3EL8	18	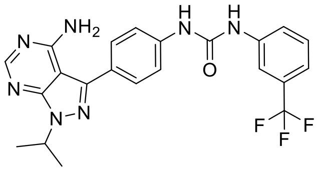	25 nM (IC_{50})	adenine pocket, DFG pocket, hydrophobic pocket II (DFG-out)	[83]
22	3G6G	19	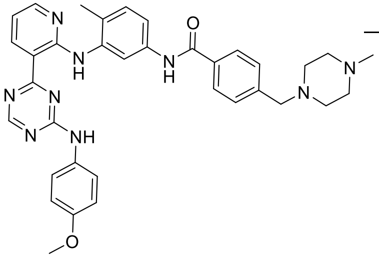	2.8 nM (IC_{50})<br>18 nM (IC_{50}) for T341I Src	adenine pocket, DFG pocket, hydrophobic pocket I and II (DFG-out)	[74]
23	3G6H (T341I)	19	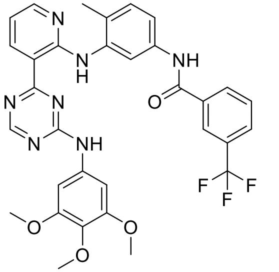	4.6 nM (IC_{50})<br>6.4 nM (IC_{50}) for T341I Src	adenine pocket, DFG pocket, hydrophobic pocket I and II (DFG-out)	[74]
24	4AGW	20	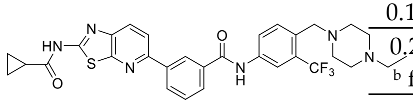	0.19 µM (EC_{50})[b]<br>0.29 µM (EC_{50}) [b] for T341I Src<br>0.15 µM (EC_{50}) [b] for T341M Src	adenine pocket, DFG pocket, hydrophobic pocket II (DFG-out)	[76]
25	3UQF	21	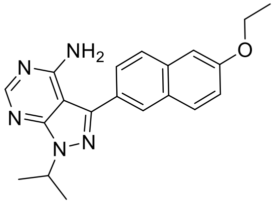	0.190 µM (IC_{50})	adenine pocket, hydrophobic pocket I and II (αC-helix-out)	[78]

27	4YBJ	22	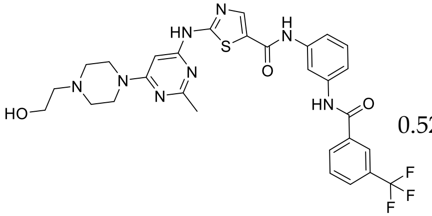	0.52 nM (K_d)	adenine pocket, DFG pocket, hydrophobic pocket I and II (DFG-out)	[69]
28	4YBK	22	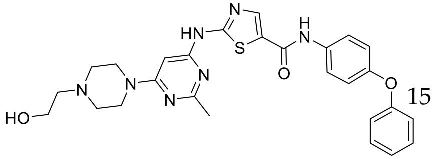	15 nM (K_d)	adenine pocket, DFG pocket, hydrophobic pocket I and II (αC-helix-out)	[69]
31	3F3V	24	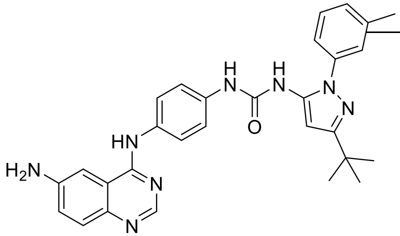	21 nM (IC_{50})	adenine pocket, DFG pocket, hydrophobic pocket II (DFG-out)	[68]
31	3F3W (T341M)	24		34 nM (IC_{50})		

[a]Binding affinities are expressed as IC_{50}, K_d or K_i values. The biological activity data for mutant c-Src are specified individually (where applicable) while the unspecified binding affinities correspond to wild-type c-Src. [b]No IC_{50}/K_d/K_i value from enzyme assays is reported; the EC_{50} values represent inhibition of Ba/F3 cells proliferation transfected with wild-type and mutant c-Src variants.

Table S4. Index of type-III inhibitors of c-Src kinase co-crystallized with the protein discussed.

Inhibitor	PDB ID	Figure	2D-Structure	Biological Activity (IC_{50})	Binding Pocket Occupancy	Ref
29	3F3U	23	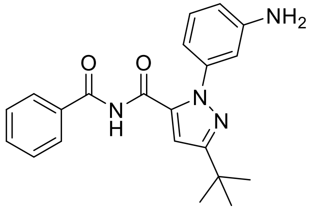	32.1 μM (IC_{50})	DFG pocket, hydrophobic pocket I	[37]
30	3F3T	23	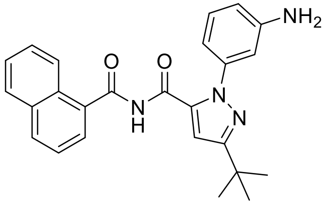	64.1 μM (IC_{50})	DFG pocket, hydrophobic pocket I	[37]

© 2020 by the authors. Licensee MDPI, Basel, Switzerland. This article is an open access article distributed under the terms and conditions of the Creative Commons Attribution (CC BY) license (http://creativecommons.org/licenses/by/4.0/).
